# Supplementary material for: VHL suppresses autophagy and tumor growth through PHD1-dependent Beclin1 hydroxylation
Source: EMBO J. 2024 Feb 15;43(6):3. doi: 10.1038/s44318-024-00051-2 (PMC10943020; doi:10.1038/s44318-024-00051-2)
Supplement: Supplementary file 1 — Appendix [file 44318_2024_51_MOESM1_ESM.pdf]

APPENDIX

VHL suppresses autophagy and tumor growth through PHD1-dependent Beclin1 hydroxylation

Zheng Wang, Meisi Yan, Leiguang Ye, Qimin Zhou, Yuran Duan, Hongfei Jiang, Lei Wang, Yuan Ouyang, Huahe Zhang, Yuli Shen, Guimei Ji, Xiaohan Chen, Qi Tian, Liwei Xiao, Qingang Wu, Ying Meng, Guijun Liu, Leina Ma, Bo Le, Zhimin Lu, Daqian Xu

TABLE OF CONTENTS

Appendix Figure S1. VHL inhibits autophagy initiation independent of its E3 ligase activity and its regulation of HIF2α expression..**page 2**

Appendix Figure S2. VHL binds to Beclin1 and inhibits the association of ATG14 with the Beclin1/VPS34/VPS15 complex..**page 4**

Appendix Figure S3. Beclin1 P54 hydroxylation inhibit glucose deprivation-induced autophagy in a VHL-dependent manner...**page 5**

Appendix Figure S4. PHD1-dependent Beclin1 hydroxylation and VHL-suppressed autophagy is not tumor type-specific...**page 7**

Appendix Figure S5. Beclin1 P54 hydroxylation-increased cell death is independent of the interaction between Beclin1 and BCL2..**page 8**

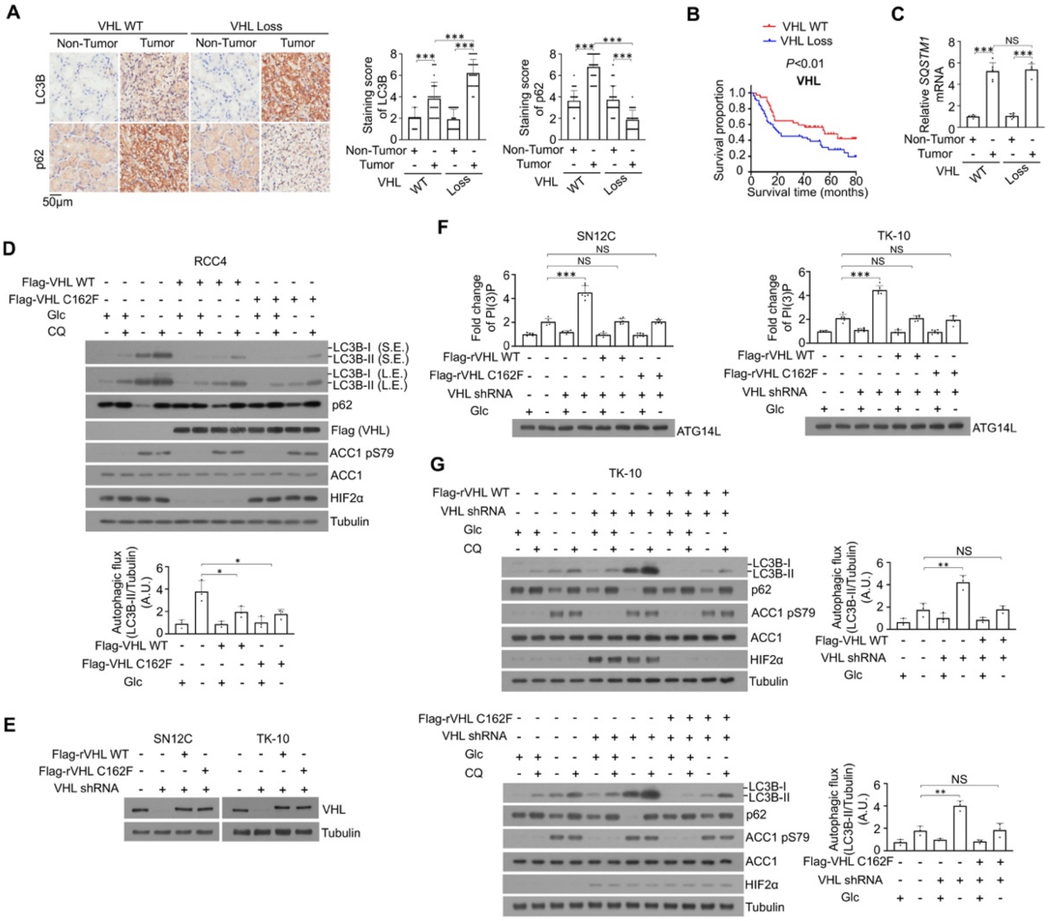

Appendix Figure S1. VHL inhibits autophagy initiation independent of its E3 ligase activity and its regulation of HIF2α expression.

(A) Representative IHC staining of human ccRCC and matched non-tumor tissue samples was performed with the indicated antibodies (left). The indicated staining scores for LC3B and p62 expression levels in ccRCC and matched non-tumor liver samples were compared (right). ***$P < 0.001$ by Mann–Whitney U test.

(B) Kaplan–Meier plots of the overall survival rates in 90 ccRCC patients with WT VHL or missense mutations. P values were calculated using a log-rank test (two-tailed).

(C) The mRNA expression levels of SQSTM1 gene were measured using quantitative PCR. ***$P < 0.001$. NS, no significance by two-tailed Student's t-test.

(D) RCC4 cells transfected with the indicated plasmids were treated with or without glucose deprivation in the presence or absence of 20 µM chloroquine (CQ) for 2 h.

(E) SN12C and TK-10 cells with and without VHL-depletion were reconstituted with VHL WT or C162F mutant.

(F) VHL-depleted SN12C and TK-10 cells reconstituted with VHL WT or C162F mutant were treated with or without glucose deprivation for 2 h. VPS34 complexes were immunoprecipitated by ATG14L antibody followed by PI(3)P detection by a quantitative ELISA. The PI(3)P level was normalized to the amount of ATG14L used in the assay.***$P < 0.001$; NS, no significance by two-tailed Student's t-test.

(G) TK-10 cells with or without VHL depletion and reconstituted expression of the indicated shRNA-resistant Flag-VHL proteins were treated with or without glucose deprivation in the presence or absence of 20 μM chloroquine (CQ) for 2 h. Autophagic flux were shown on the right. Data information: Data represent the mean ± SD. Means were compared using indicated statistical method. **D, E, G,** immunoblotting analyses were performed. Experiments were repeated at least twice with similar results.

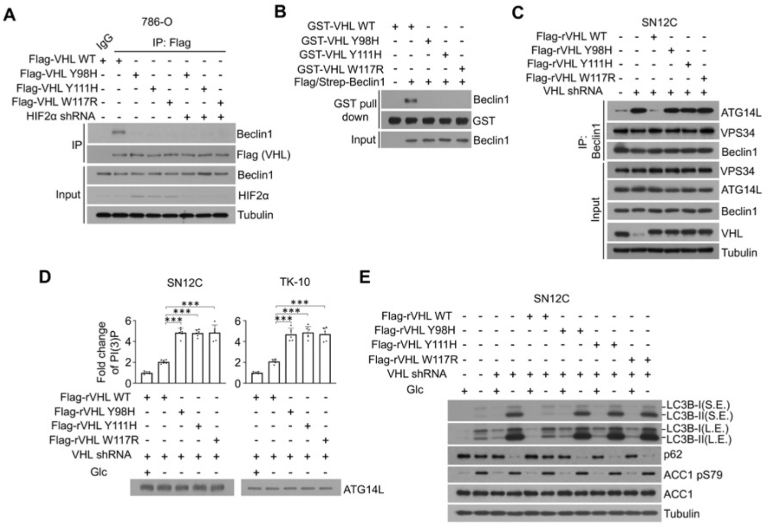

Appendix Figure S2. VHL binds to Beclin1 and inhibits the association of ATG14 with the Beclin1/VPS34/VPS15 complex.

(A) 786-O cells stably transfected with the indicated Flag-VHL constructs and shRNA were harvested for immunoprecipitation and immunoblotting analyses as indicated.

(B) A GST pull down assay was performed by mixing purified GST-VHL and different Flag/Strep-tagged Beclin1 proteins purified from 293T cells. Immunoblotting analyses were performed as indicated.

(C) Endogenous VHL-depleted SN12C cells were reconstituted with different shRNA-resistant VHL and harvested for immunoprecipitation and immunoblotting analyses as indicated.

(D) Endogenous VHL-depleted SN12C or TK-10 cells reconstituted with different shRNA-resistant VHL were treated with or without glucose deprivation for 2 h. VPS34 complexes were immunoprecipitated by ATG14L antibody followed by PI(3)P detection by the quantitative ELISA. The PI(3)P level was normalized to the amount of ATG14L used in the assay.

(E) Endogenous VHL-depleted SN12C cells reconstituted with different shRNA-resistant VHL were treated with or without glucose deprivation for 2 h. The cell lysates were harvested for immunoblotting analyses were performed as indicated.

Data information: Data represent the mean ± SD. The statistical significance was determined using two-tailed Student's t-test. ***P < 0.001. All experiments were repeated three times with similar results.

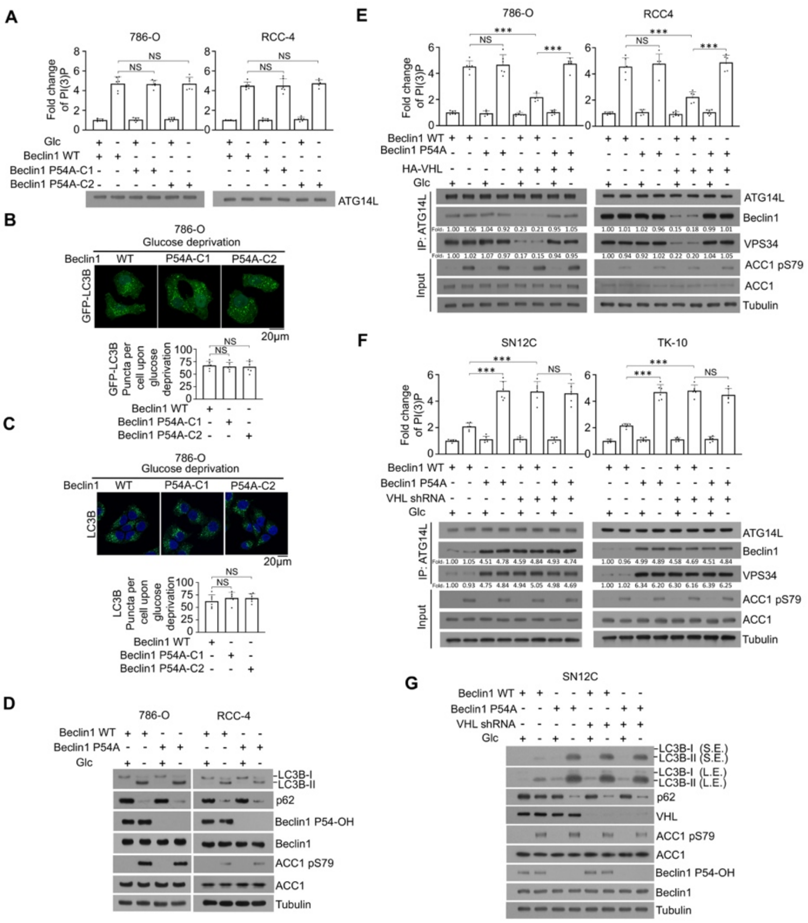

Appendix Figure S3. Beclin1 P54 hydroxylation inhibit glucose deprivation-induced autophagy in a VHL-dependent manner.

(A) Parental 786-O and RCC-4 cells and the indicated clones with knock-in expression of Beclin1 P54A were treated with glucose deprivation for 2 h. VPS34 complexes were immunoprecipitated by ATG14L antibody followed by PI(3)P detection by quantitative ELISA. The PI(3)P level was normalized to the amount of ATG14L used in the assay.

(B, C) Parental 786-O cells and the indicated clones with knock-in expression of Beclin1 P54A were treated with glucose deprivation for 2 h. Representative images of GFP-LC3B (B) or endogenous LC3B (C) puncta are shown (upper). The numbers of LC3B puncta were quantitated (lower).

(D) Parental 786-O and RCC-4 cells and the indicated clones with knock-in expression of Beclin1 P54A were treated with or without glucose deprivation for 2 h.

(E) Parental 786-O and RCC4 cells as well as the indicated clones with knock-in expression of Beclin1 P54A were transfected with HA-VHL and treated with or without glucose deprivation for 2 h. VPS34 complexes were immunoprecipitated by ATG14L antibody followed by PI(3)P detection by the quantitative ELISA. The PI(3)P level was normalized to the amount of ATG14L used in the assay. Relative fold changes of Beclin1 and VPS34 were shown.

(F) Parental SN12C and TK-10 cells and the indicated clones with knock-in expression of Beclin1 P54A were stably transfected with or without VHL shRNA and treated with glucose deprivation for 2 h. VPS34 complexes were immunoprecipitated by ATG14L antibody followed by PI(3)P detection by quantitative ELISA. The PI(3)P level was normalized to the amount of ATG14L used in the assay.

Relative fold changes of Beclin1 and VPS34 were shown.

(G) Parental SN12C cells and the indicated clone with knock-in expression of Beclin1 P54A were stably transfected with or without VHL shRNA and treated with glucose deprivation for 2 h. The whole cell lysates were harvested for immunoblotting analyses as indicated.

Data information: Data represent the mean ± SD. The statistical significance was determined using two-tailed Student's t-test. ***$P < 0.001$. C1, clone 1; C2, clone 2. All experiments were repeated at least twice with similar results.

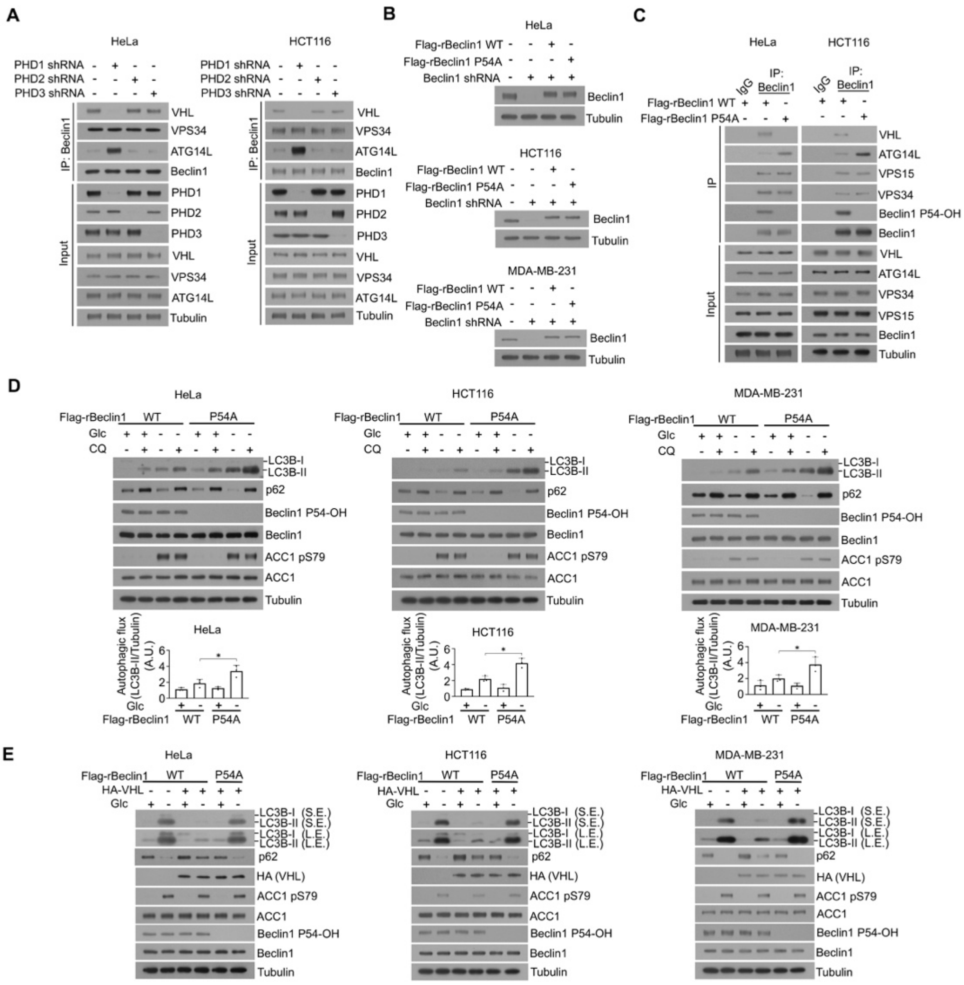

Appendix Figure S4. PHD1-dependent Beclin1 hydroxylation and VHL-suppressed autophagy is not tumor type-specific.

(A) HeLa (transfected with LKB1) and HCT116 cells stably transfected with the indicated shRNA were harvested for immunoprecipitation and immunoblotting analyses as indicated.

(B, C) Endogenous Beclin1 depleted HeLa (transfected with LKB1), HCT116 or MDA-MB-231 cells were stably transfected with the indicated shRNA-resistant Flag-Beclin1 proteins.

(D, E) Endogenous Beclin1 depleted HeLa (transfected with LKB1), HCT116 or MDA-MB-231 cells stably transfected with the indicated constructs were treated with or without glucose deprivation in the presence or absence of 20 µM chloroquine (CQ) for 4 h. Autophagic flux were shown (D).

Data information: Immunoprecipitation and/or immunoblotting analyses were performed as indicated. All experiments were repeated three times with similar results.

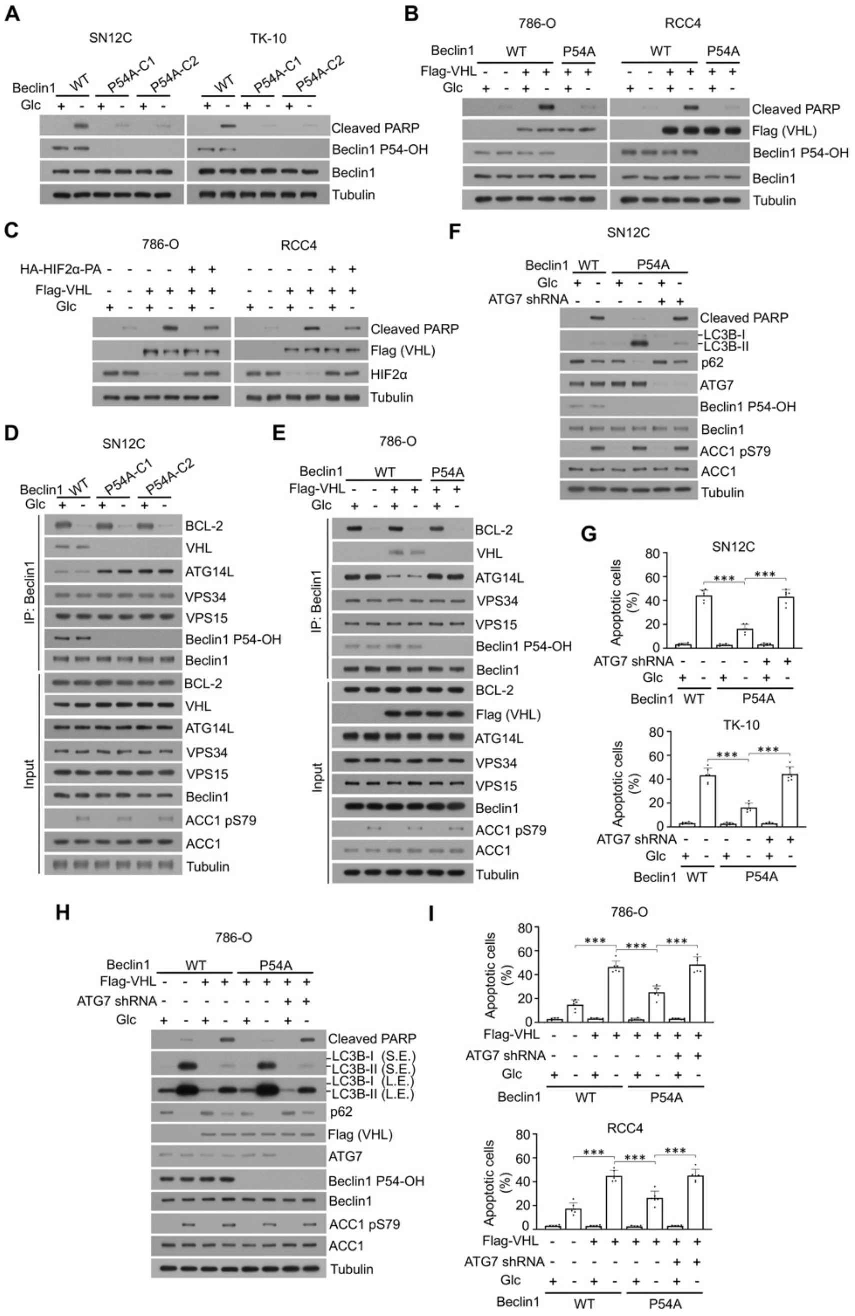

Appendix Figure S5. Beclin1 P54 hydroxylation-increased cell death is independent of the interaction between Beclin1 and BCL2.

(A) Parental SN12C and TK-10 cells and the indicated clones with Beclin1 P54A knock-in expression were treated with or without glucose deprivation for 8 h. The whole cell lysates were harvested for immunoblotting analyses as indicated.

(B, C) Parental 786-O and RCC4 cells and the indicated clones with Beclin1 P54A knock-in expression were stably transfected with the indicated plasmid and treated with or without glucose deprivation for 8 h. The whole cell lysates were harvested for immunoblotting analyses as indicated.

(D) Parental SN12C cells and the indicated clones with Beclin1 P54A knock-in expression were treated with or without glucose deprivation for 2 h. The whole cell lysates were harvested for immunoprecipitation and immunoblotting analyses as indicated.

(E) Parental 786-O cells and the indicated clones with Beclin1 P54A knock-in expression

transfected with or without the constructs were treated with or without glucose deprivation for 2 h. The whole cell lysates were harvested for immunoprecipitation and immunoblotting analyses as indicated.

(F) Parental SN12C cells and the indicated clones with Beclin1 P54A knock-in expression stably transfected with or without ATG7 shRNA were treated with or without glucose deprivation for 8 h. The whole cell lysates were harvested for immunoblotting analyses as indicated.

(G) Parental SN12C and TK-10 cells and the indicated clones with Beclin1 P54A knock-in expression stably transfected with or without ATG7 shRNA were treated with or without glucose deprivation for 8 h. Apoptotic cells were counted.

(H) Parental 786-O and the indicated clones with Beclin1 P54A knock-in expression stably transfected with the indicated constructs were treated with or without glucose deprivation for 8 h. The whole cell lysates were harvested for immunoblotting analyses as indicated.

(I) Parental 786-O and RCC4 cells and the indicated clones with Beclin1 P54A knock-in expression stably transfected with the indicated constructs were treated with or without glucose deprivation for 8 h. Apoptotic cells were counted.

Data information: Data represent the mean ± SD. The statistical significance was determined using two-tailed Student's t-test. ***$P < 0.001$. C1, clone 1; C2, clone 2. All experiments were repeated at least twice with similar results.
