# Supplementary figures and images for: VHL suppresses autophagy and tumor growth through PHD1-dependent Beclin1 hydroxylation (part 1 of 2)
Source: EMBO J. 2024 Feb 15;43(6):3. doi: 10.1038/s44318-024-00051-2 (PMC10943020; doi:10.1038/s44318-024-00051-2)

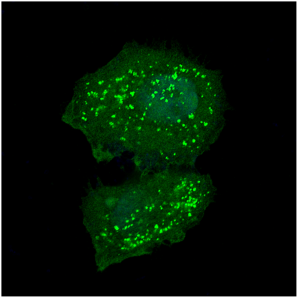

Supplement: Supplementary file 2 — Source Data Fig. 1 [file 44318_2024_51_MOESM2_ESM.zip › SD Fig 1/Fig 1D/Glc-.tiff]

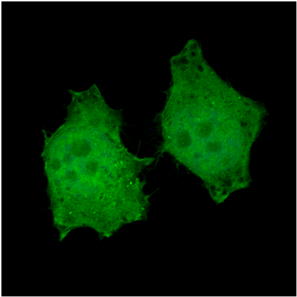

Supplement: Supplementary file 2 — Source Data Fig. 1 [file 44318_2024_51_MOESM2_ESM.zip › SD Fig 1/Fig 1D/Glc+.tiff]

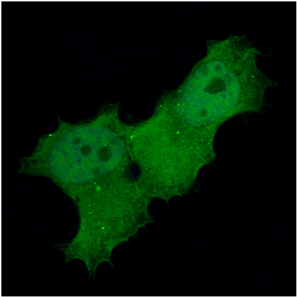

Supplement: Supplementary file 2 — Source Data Fig. 1 [file 44318_2024_51_MOESM2_ESM.zip › SD Fig 1/Fig 1D/VHL WT Glc-.tiff]

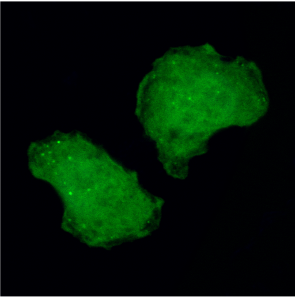

Supplement: Supplementary file 2 — Source Data Fig. 1 [file 44318_2024_51_MOESM2_ESM.zip › SD Fig 1/Fig 1D/VHL C162F Glc-.tiff]

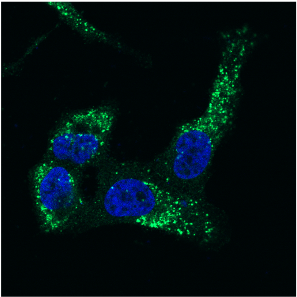

Supplement: Supplementary file 2 — Source Data Fig. 1 [file 44318_2024_51_MOESM2_ESM.zip › SD Fig 1/Fig 1E/Glc-.tiff]

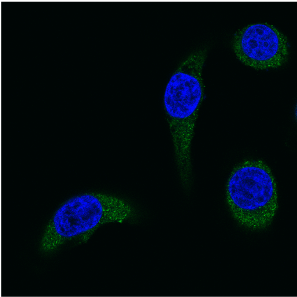

Supplement: Supplementary file 2 — Source Data Fig. 1 [file 44318_2024_51_MOESM2_ESM.zip › SD Fig 1/Fig 1E/Glc+.tiff]

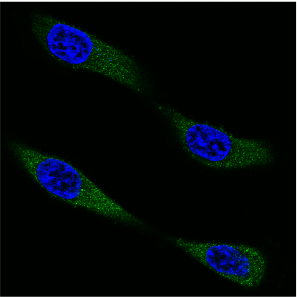

Supplement: Supplementary file 2 — Source Data Fig. 1 [file 44318_2024_51_MOESM2_ESM.zip › SD Fig 1/Fig 1E/VHL WT Glc-.tiff]

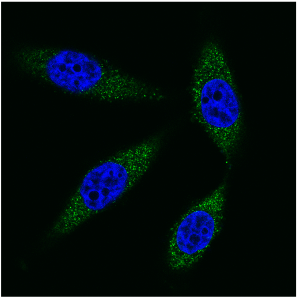

Supplement: Supplementary file 2 — Source Data Fig. 1 [file 44318_2024_51_MOESM2_ESM.zip › SD Fig 1/Fig 1E/VHL C162F Glc-.tiff]

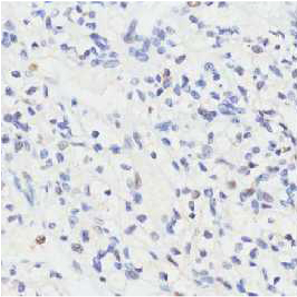

Supplement: Supplementary file 2 — Source Data Fig. 1 [file 44318_2024_51_MOESM2_ESM.zip › SD Fig 1/Fig 1A/VHL loss/Tumor5 VHL.tiff]

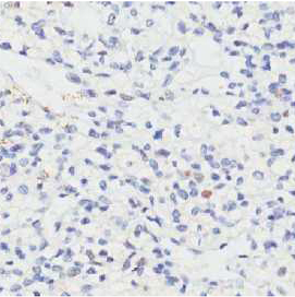

Supplement: Supplementary file 2 — Source Data Fig. 1 [file 44318_2024_51_MOESM2_ESM.zip › SD Fig 1/Fig 1A/VHL loss/Tumor4 VHL.tiff]

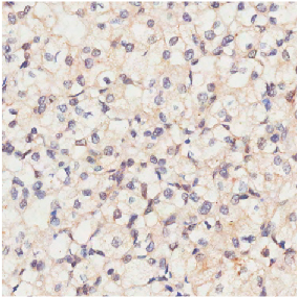

Supplement: Supplementary file 2 — Source Data Fig. 1 [file 44318_2024_51_MOESM2_ESM.zip › SD Fig 1/Fig 1A/VHL loss/Tumor6 p62.tiff]

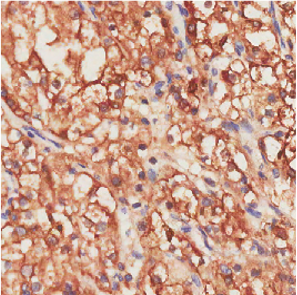

Supplement: Supplementary file 2 — Source Data Fig. 1 [file 44318_2024_51_MOESM2_ESM.zip › SD Fig 1/Fig 1A/VHL loss/Tumor6 LC3B.tiff]

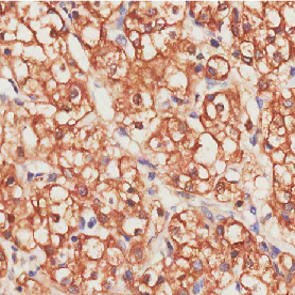

Supplement: Supplementary file 2 — Source Data Fig. 1 [file 44318_2024_51_MOESM2_ESM.zip › SD Fig 1/Fig 1A/VHL loss/Tumor5 LC3B.tiff]

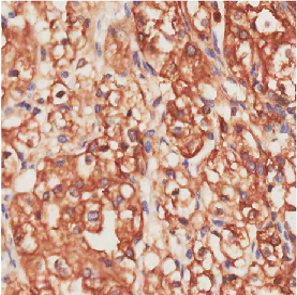

Supplement: Supplementary file 2 — Source Data Fig. 1 [file 44318_2024_51_MOESM2_ESM.zip › SD Fig 1/Fig 1A/VHL loss/Tumor4 LC3B.tiff]

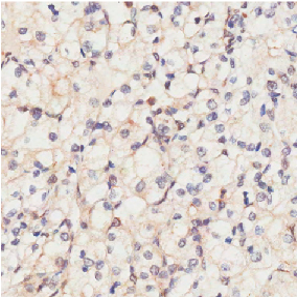

Supplement: Supplementary file 2 — Source Data Fig. 1 [file 44318_2024_51_MOESM2_ESM.zip › SD Fig 1/Fig 1A/VHL loss/Tumor5 p62.tiff]

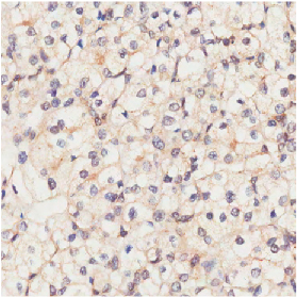

Supplement: Supplementary file 2 — Source Data Fig. 1 [file 44318_2024_51_MOESM2_ESM.zip › SD Fig 1/Fig 1A/VHL loss/Tumor4 p62.tiff]

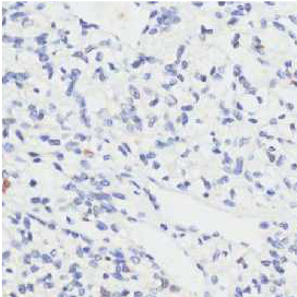

Supplement: Supplementary file 2 — Source Data Fig. 1 [file 44318_2024_51_MOESM2_ESM.zip › SD Fig 1/Fig 1A/VHL loss/Tumor6 VHL.tiff]

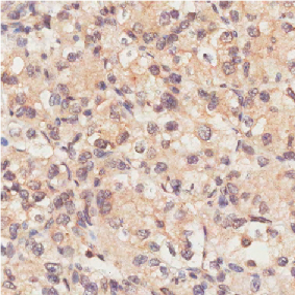

Supplement: Supplementary file 2 — Source Data Fig. 1 [file 44318_2024_51_MOESM2_ESM.zip › SD Fig 1/Fig 1A/VHL WT/Tumor1 LC3B.tiff]

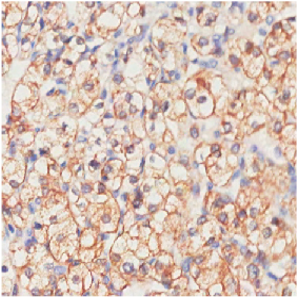

Supplement: Supplementary file 2 — Source Data Fig. 1 [file 44318_2024_51_MOESM2_ESM.zip › SD Fig 1/Fig 1A/VHL WT/Tumor2 VHL.tiff]

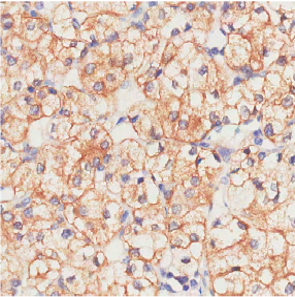

Supplement: Supplementary file 2 — Source Data Fig. 1 [file 44318_2024_51_MOESM2_ESM.zip › SD Fig 1/Fig 1A/VHL WT/Tumor3 VHL.tiff]

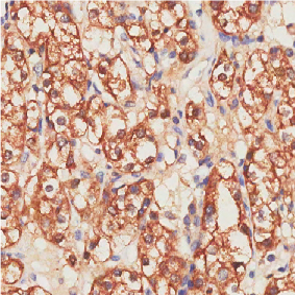

Supplement: Supplementary file 2 — Source Data Fig. 1 [file 44318_2024_51_MOESM2_ESM.zip › SD Fig 1/Fig 1A/VHL WT/Tumor1 p62.tiff]

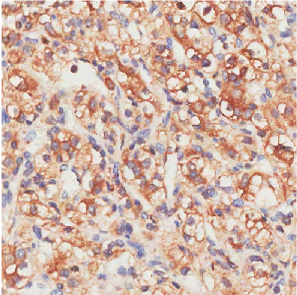

Supplement: Supplementary file 2 — Source Data Fig. 1 [file 44318_2024_51_MOESM2_ESM.zip › SD Fig 1/Fig 1A/VHL WT/Tumor2 p62.tiff]

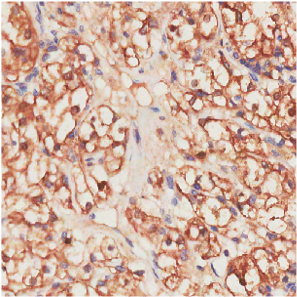

Supplement: Supplementary file 2 — Source Data Fig. 1 [file 44318_2024_51_MOESM2_ESM.zip › SD Fig 1/Fig 1A/VHL WT/Tumor3 p62.tiff]

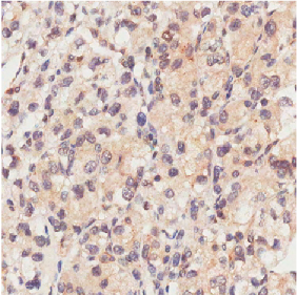

Supplement: Supplementary file 2 — Source Data Fig. 1 [file 44318_2024_51_MOESM2_ESM.zip › SD Fig 1/Fig 1A/VHL WT/Tumor3 LC3B.tiff]

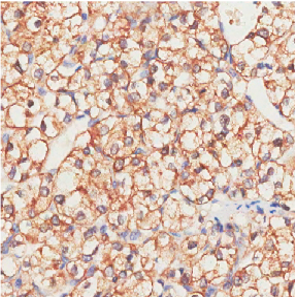

Supplement: Supplementary file 2 — Source Data Fig. 1 [file 44318_2024_51_MOESM2_ESM.zip › SD Fig 1/Fig 1A/VHL WT/Tumor1 VHL.tiff]

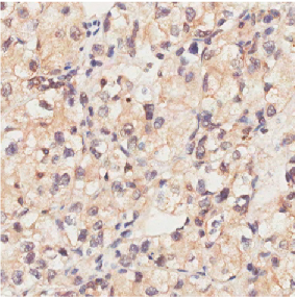

Supplement: Supplementary file 2 — Source Data Fig. 1 [file 44318_2024_51_MOESM2_ESM.zip › SD Fig 1/Fig 1A/VHL WT/Tumor2 LC3B.tiff]

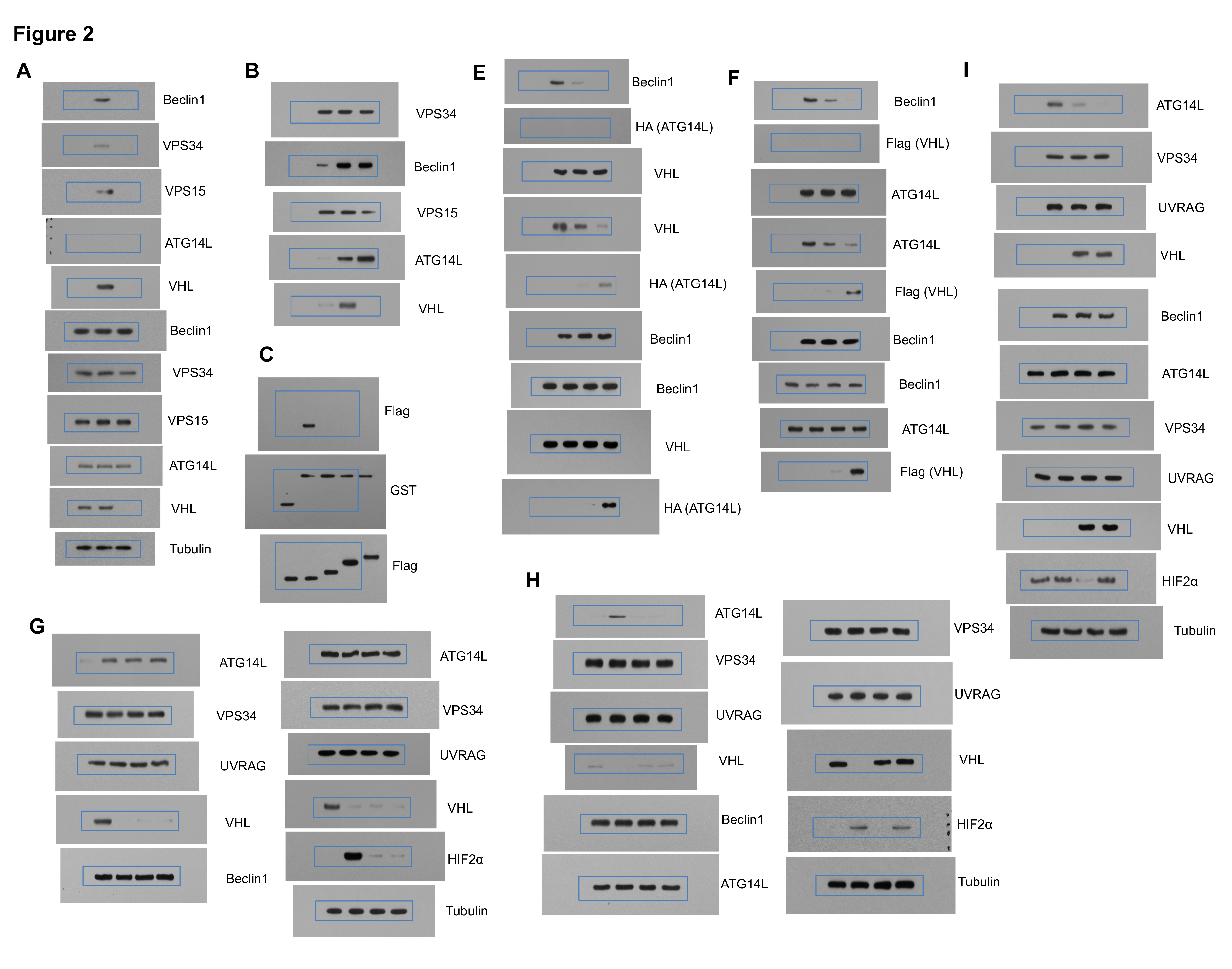

Supplement: Supplementary file 3 — Source Data Fig. 2 [file 44318_2024_51_MOESM3_ESM.zip › SD Fig 2/SD-Fig 2.jpg]

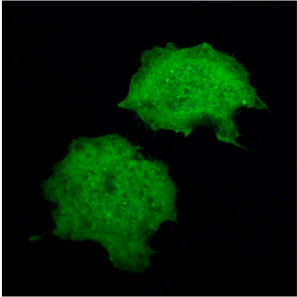

Supplement: Supplementary file 4 — Source Data Fig. 3 [file 44318_2024_51_MOESM4_ESM.zip › SD Fig 3/Fig 3C/Glc- VHL WT.tiff]

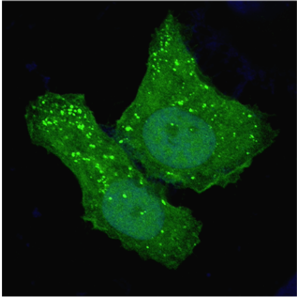

Supplement: Supplementary file 4 — Source Data Fig. 3 [file 44318_2024_51_MOESM4_ESM.zip › SD Fig 3/Fig 3C/Glc- VHL Y98H.tiff]

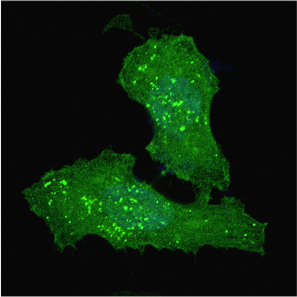

Supplement: Supplementary file 4 — Source Data Fig. 3 [file 44318_2024_51_MOESM4_ESM.zip › SD Fig 3/Fig 3C/Glc- mock.tiff]

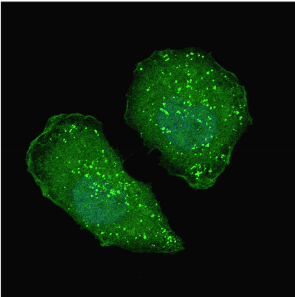

Supplement: Supplementary file 4 — Source Data Fig. 3 [file 44318_2024_51_MOESM4_ESM.zip › SD Fig 3/Fig 3C/Glc- VHL W117R.tiff]

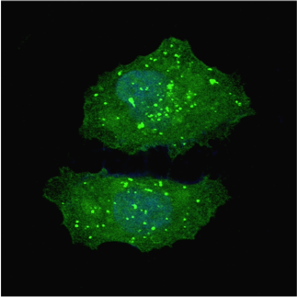

Supplement: Supplementary file 4 — Source Data Fig. 3 [file 44318_2024_51_MOESM4_ESM.zip › SD Fig 3/Fig 3C/Glc- VHL Y111H.tiff]

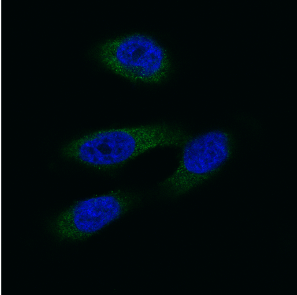

Supplement: Supplementary file 4 — Source Data Fig. 3 [file 44318_2024_51_MOESM4_ESM.zip › SD Fig 3/Fig 3D/Glc- VHLWT.tiff]

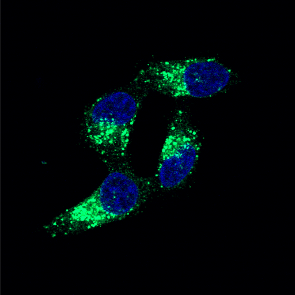

Supplement: Supplementary file 4 — Source Data Fig. 3 [file 44318_2024_51_MOESM4_ESM.zip › SD Fig 3/Fig 3D/Glc- VHL Y98H.tiff]

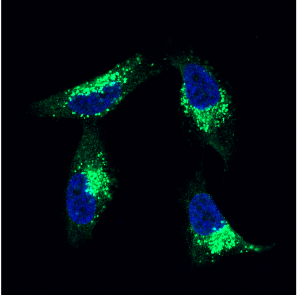

Supplement: Supplementary file 4 — Source Data Fig. 3 [file 44318_2024_51_MOESM4_ESM.zip › SD Fig 3/Fig 3D/Glc- mock.tiff]

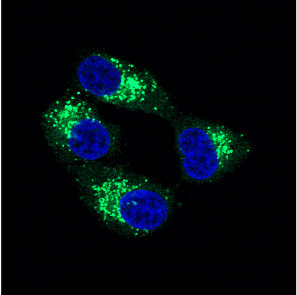

Supplement: Supplementary file 4 — Source Data Fig. 3 [file 44318_2024_51_MOESM4_ESM.zip › SD Fig 3/Fig 3D/Glc- VHL W117R.tiff]

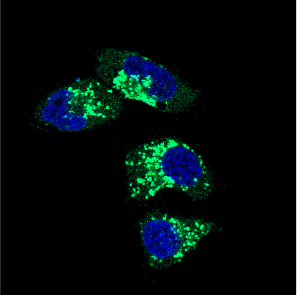

Supplement: Supplementary file 4 — Source Data Fig. 3 [file 44318_2024_51_MOESM4_ESM.zip › SD Fig 3/Fig 3D/Glc- VHL Y111H.tiff]

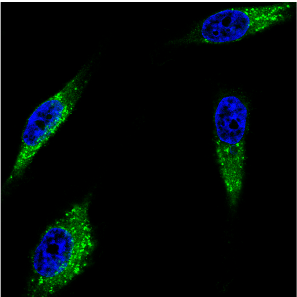

Supplement: Supplementary file 4 — Source Data Fig. 3 [file 44318_2024_51_MOESM4_ESM.zip › SD Fig 3/Fig 3J/Glc- shPHD1.tiff]

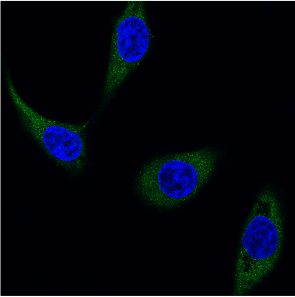

Supplement: Supplementary file 4 — Source Data Fig. 3 [file 44318_2024_51_MOESM4_ESM.zip › SD Fig 3/Fig 3J/Glc- shPHD3.tiff]

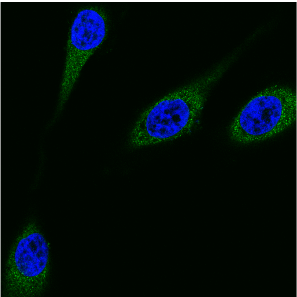

Supplement: Supplementary file 4 — Source Data Fig. 3 [file 44318_2024_51_MOESM4_ESM.zip › SD Fig 3/Fig 3J/Glc- shPHD2.tiff]

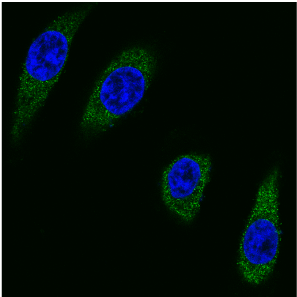

Supplement: Supplementary file 4 — Source Data Fig. 3 [file 44318_2024_51_MOESM4_ESM.zip › SD Fig 3/Fig 3J/Glc- Mock.tiff]

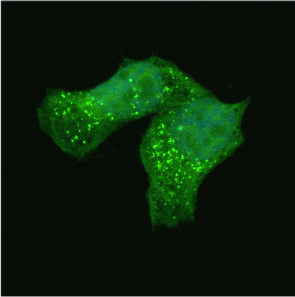

Supplement: Supplementary file 4 — Source Data Fig. 3 [file 44318_2024_51_MOESM4_ESM.zip › SD Fig 3/Fig 3I/Glc- shPHD1.tiff]

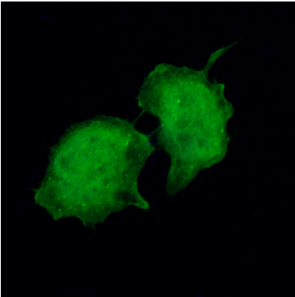

Supplement: Supplementary file 4 — Source Data Fig. 3 [file 44318_2024_51_MOESM4_ESM.zip › SD Fig 3/Fig 3I/Glc- shPHD3.tiff]

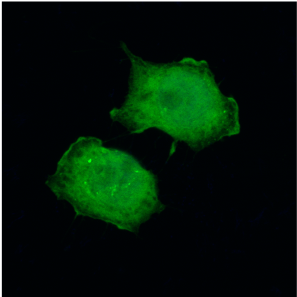

Supplement: Supplementary file 4 — Source Data Fig. 3 [file 44318_2024_51_MOESM4_ESM.zip › SD Fig 3/Fig 3I/Glc- shPHD2.tiff]

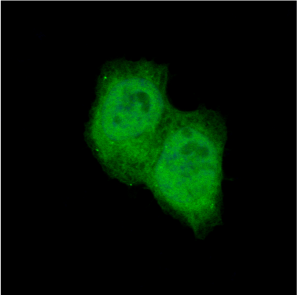

Supplement: Supplementary file 4 — Source Data Fig. 3 [file 44318_2024_51_MOESM4_ESM.zip › SD Fig 3/Fig 3I/Glc- mock.tiff]

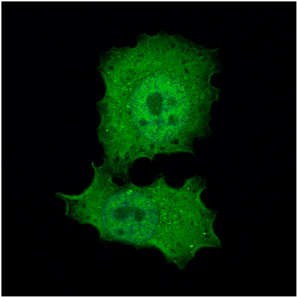

Supplement: Supplementary file 5 — Source Data Fig. 4 [file 44318_2024_51_MOESM5_ESM.zip › SD Fig 4/Fig 4J/Glc- Beclin1 WT+VHL.tiff]

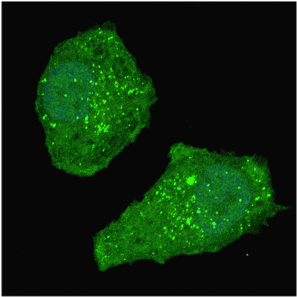

Supplement: Supplementary file 5 — Source Data Fig. 4 [file 44318_2024_51_MOESM5_ESM.zip › SD Fig 4/Fig 4J/Glc- Beclin1 WT.tiff]

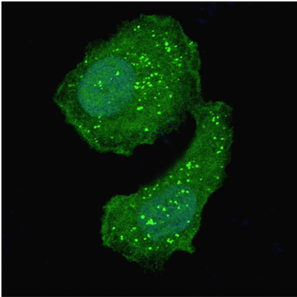

Supplement: Supplementary file 5 — Source Data Fig. 4 [file 44318_2024_51_MOESM5_ESM.zip › SD Fig 4/Fig 4J/Glc- Beclin1 P54A+VHL.tiff]

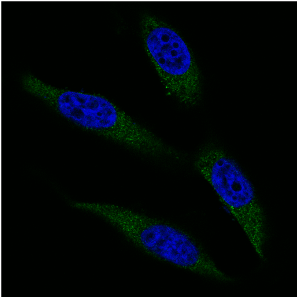

Supplement: Supplementary file 5 — Source Data Fig. 4 [file 44318_2024_51_MOESM5_ESM.zip › SD Fig 4/Fig 4K/Glc- Beclin1 WT+VHL.tiff]

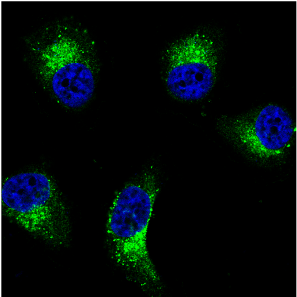

Supplement: Supplementary file 5 — Source Data Fig. 4 [file 44318_2024_51_MOESM5_ESM.zip › SD Fig 4/Fig 4K/Glc- Beclin1 WT.tiff]

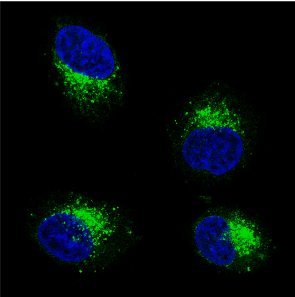

Supplement: Supplementary file 5 — Source Data Fig. 4 [file 44318_2024_51_MOESM5_ESM.zip › SD Fig 4/Fig 4K/Glc- Beclin1 P54A+VHL.tiff]

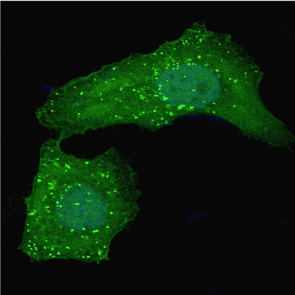

Supplement: Supplementary file 5 — Source Data Fig. 4 [file 44318_2024_51_MOESM5_ESM.zip › SD Fig 4/Fig 4G/Glc- Beclin1 P54A-C1.tiff]

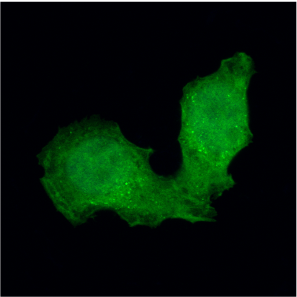

Supplement: Supplementary file 5 — Source Data Fig. 4 [file 44318_2024_51_MOESM5_ESM.zip › SD Fig 4/Fig 4G/Glc- Beclin1 WT.tiff]

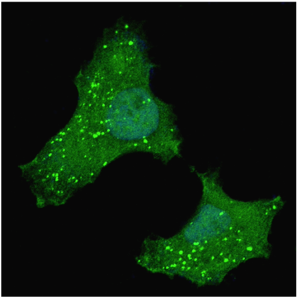

Supplement: Supplementary file 5 — Source Data Fig. 4 [file 44318_2024_51_MOESM5_ESM.zip › SD Fig 4/Fig 4G/Glc- Beclin1 P54A-C2.tiff]

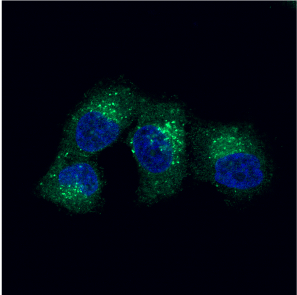

Supplement: Supplementary file 5 — Source Data Fig. 4 [file 44318_2024_51_MOESM5_ESM.zip › SD Fig 4/Fig 4H/Glc- Beclin1 P54A-C1.tiff]

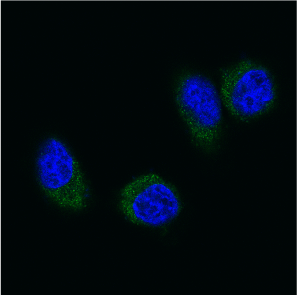

Supplement: Supplementary file 5 — Source Data Fig. 4 [file 44318_2024_51_MOESM5_ESM.zip › SD Fig 4/Fig 4H/Glc- Beclin1 WT.tiff]

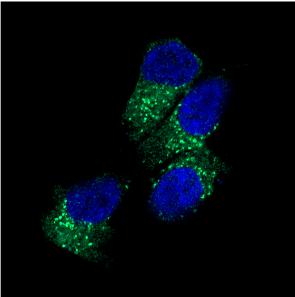

Supplement: Supplementary file 5 — Source Data Fig. 4 [file 44318_2024_51_MOESM5_ESM.zip › SD Fig 4/Fig 4H/Glc- Beclin1 P54A-C2.tiff]

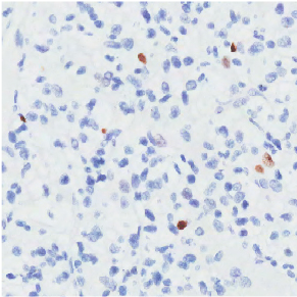

Supplement: Supplementary file 6 — Source Data Fig. 5 [file 44318_2024_51_MOESM6_ESM.zip › SD Fig 5/Fig 5I/mock.tiff]

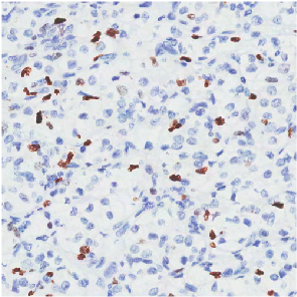

Supplement: Supplementary file 6 — Source Data Fig. 5 [file 44318_2024_51_MOESM6_ESM.zip › SD Fig 5/Fig 5I/PT2385.tiff]

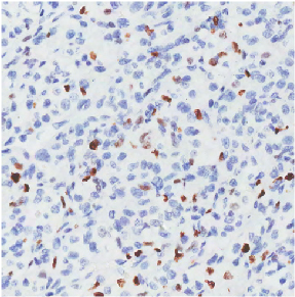

Supplement: Supplementary file 6 — Source Data Fig. 5 [file 44318_2024_51_MOESM6_ESM.zip › SD Fig 5/Fig 5I/SAR405.tiff]

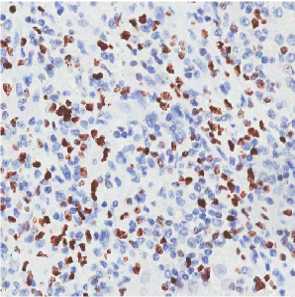

Supplement: Supplementary file 6 — Source Data Fig. 5 [file 44318_2024_51_MOESM6_ESM.zip › SD Fig 5/Fig 5I/PT2385+SAR405.tiff]

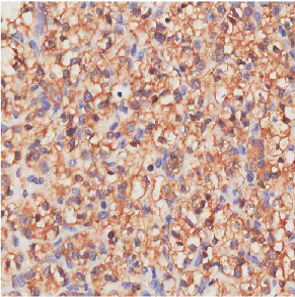

Supplement: Supplementary file 6 — Source Data Fig. 5 [file 44318_2024_51_MOESM6_ESM.zip › SD Fig 5/Fig 5G/Beclin1 WT LC3B.tiff]

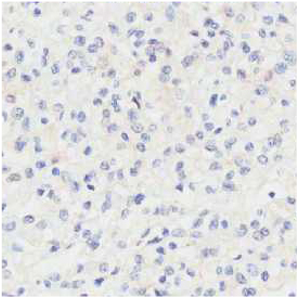

Supplement: Supplementary file 6 — Source Data Fig. 5 [file 44318_2024_51_MOESM6_ESM.zip › SD Fig 5/Fig 5G/Beclin1 P54A P54-OH.tiff]

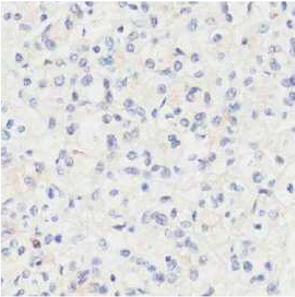

Supplement: Supplementary file 6 — Source Data Fig. 5 [file 44318_2024_51_MOESM6_ESM.zip › SD Fig 5/Fig 5G/Beclin1 WT p62.tiff]

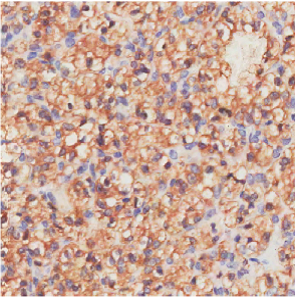

Supplement: Supplementary file 6 — Source Data Fig. 5 [file 44318_2024_51_MOESM6_ESM.zip › SD Fig 5/Fig 5G/VHL+Beclin1 WT p62.tiff]

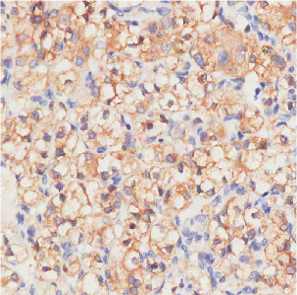

Supplement: Supplementary file 6 — Source Data Fig. 5 [file 44318_2024_51_MOESM6_ESM.zip › SD Fig 5/Fig 5G/VHL+Beclin1 P54A VHL.tiff]

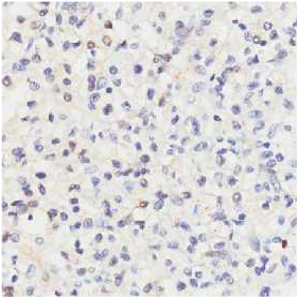

Supplement: Supplementary file 6 — Source Data Fig. 5 [file 44318_2024_51_MOESM6_ESM.zip › SD Fig 5/Fig 5G/Beclin1 P54A VHL.tiff]

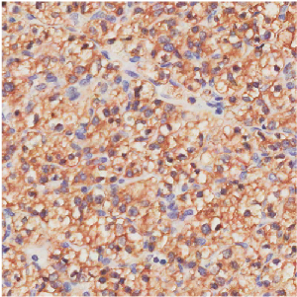

Supplement: Supplementary file 6 — Source Data Fig. 5 [file 44318_2024_51_MOESM6_ESM.zip › SD Fig 5/Fig 5G/Beclin1 P54A LC3B.tiff]

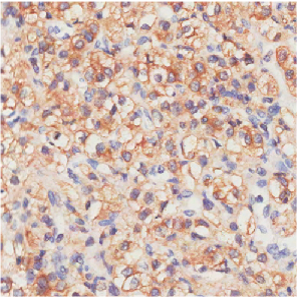

Supplement: Supplementary file 6 — Source Data Fig. 5 [file 44318_2024_51_MOESM6_ESM.zip › SD Fig 5/Fig 5G/VHL+Beclin1 WT P54-OH.tiff]

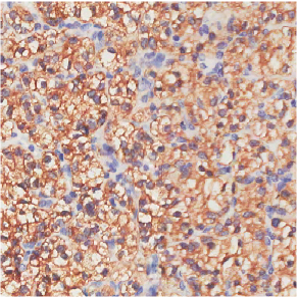

Supplement: Supplementary file 6 — Source Data Fig. 5 [file 44318_2024_51_MOESM6_ESM.zip › SD Fig 5/Fig 5G/VHL+Beclin1 P54A LC3B.tiff]

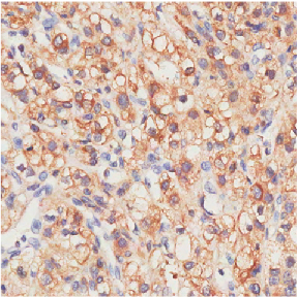

Supplement: Supplementary file 6 — Source Data Fig. 5 [file 44318_2024_51_MOESM6_ESM.zip › SD Fig 5/Fig 5G/Beclin1 WT P54-OH.tiff]

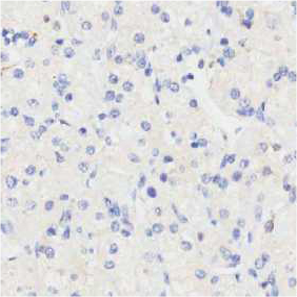

Supplement: Supplementary file 6 — Source Data Fig. 5 [file 44318_2024_51_MOESM6_ESM.zip › SD Fig 5/Fig 5G/VHL+Beclin1 P54A P54-OH.tiff]

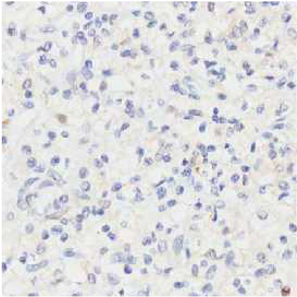

Supplement: Supplementary file 6 — Source Data Fig. 5 [file 44318_2024_51_MOESM6_ESM.zip › SD Fig 5/Fig 5G/Beclin1 P54A p62.tiff]

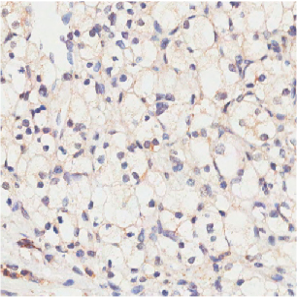

Supplement: Supplementary file 6 — Source Data Fig. 5 [file 44318_2024_51_MOESM6_ESM.zip › SD Fig 5/Fig 5G/VHL+Beclin1 P54A p62.tiff]

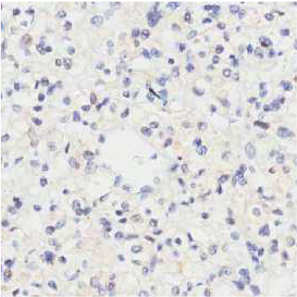

Supplement: Supplementary file 6 — Source Data Fig. 5 [file 44318_2024_51_MOESM6_ESM.zip › SD Fig 5/Fig 5G/Beclin1 WT VHL.tiff]

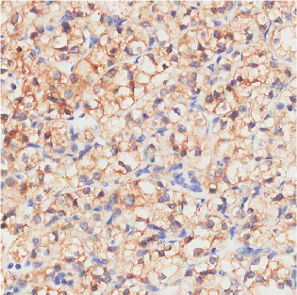

Supplement: Supplementary file 6 — Source Data Fig. 5 [file 44318_2024_51_MOESM6_ESM.zip › SD Fig 5/Fig 5G/VHL+Beclin1 WT VHL.tiff]

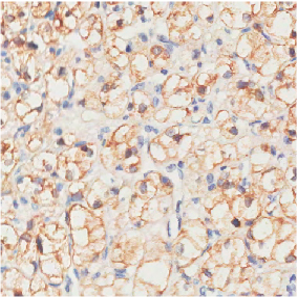

Supplement: Supplementary file 6 — Source Data Fig. 5 [file 44318_2024_51_MOESM6_ESM.zip › SD Fig 5/Fig 5G/VHL+Beclin1 WT LC3B.tiff]

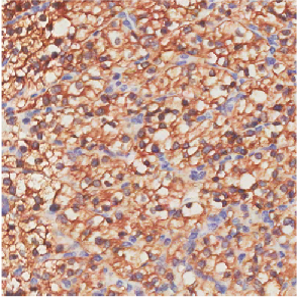

Supplement: Supplementary file 6 — Source Data Fig. 5 [file 44318_2024_51_MOESM6_ESM.zip › SD Fig 5/Fig 5E/shVHL+Beclin1 WT LC3B.tiff]

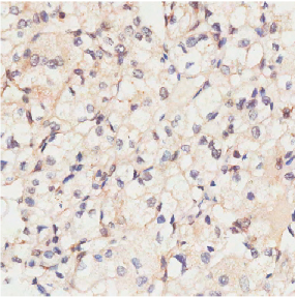

Supplement: Supplementary file 6 — Source Data Fig. 5 [file 44318_2024_51_MOESM6_ESM.zip › SD Fig 5/Fig 5E/shVHL+Beclin1 WT VHL.tiff]

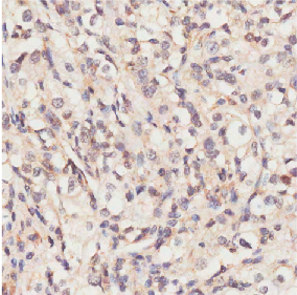

Supplement: Supplementary file 6 — Source Data Fig. 5 [file 44318_2024_51_MOESM6_ESM.zip › SD Fig 5/Fig 5E/Beclin1 WT LC3B.tiff]

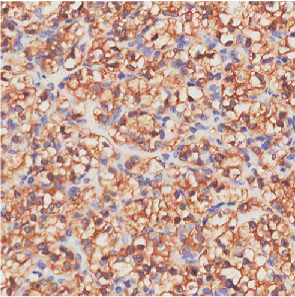

Supplement: Supplementary file 6 — Source Data Fig. 5 [file 44318_2024_51_MOESM6_ESM.zip › SD Fig 5/Fig 5E/shVHL+Beclin1 P54A LC3B.tiff]

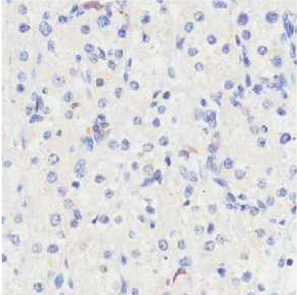

Supplement: Supplementary file 6 — Source Data Fig. 5 [file 44318_2024_51_MOESM6_ESM.zip › SD Fig 5/Fig 5E/Beclin1 P54A P54-OH.tiff]

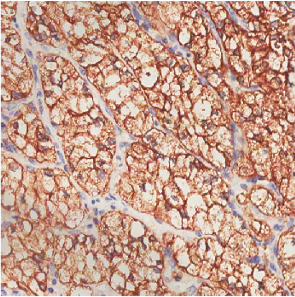

Supplement: Supplementary file 6 — Source Data Fig. 5 [file 44318_2024_51_MOESM6_ESM.zip › SD Fig 5/Fig 5E/Beclin1 WT p62.tiff]

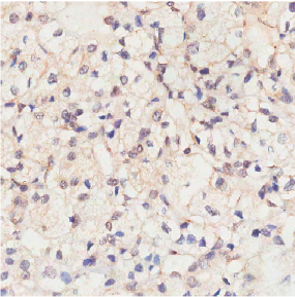

Supplement: Supplementary file 6 — Source Data Fig. 5 [file 44318_2024_51_MOESM6_ESM.zip › SD Fig 5/Fig 5E/shVHL+Beclin1 P54A VHL.tiff]

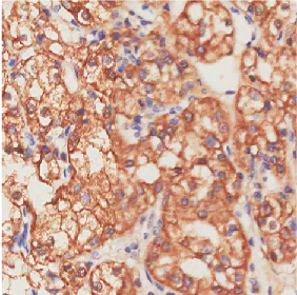

Supplement: Supplementary file 6 — Source Data Fig. 5 [file 44318_2024_51_MOESM6_ESM.zip › SD Fig 5/Fig 5E/Beclin1 P54A VHL.tiff]

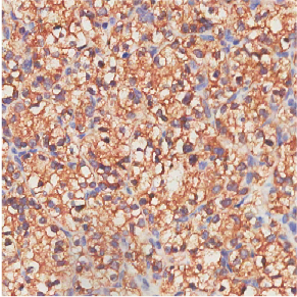

Supplement: Supplementary file 6 — Source Data Fig. 5 [file 44318_2024_51_MOESM6_ESM.zip › SD Fig 5/Fig 5E/Beclin1 P54A LC3B.tiff]

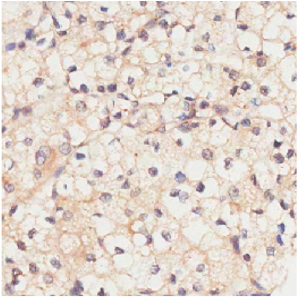

Supplement: Supplementary file 6 — Source Data Fig. 5 [file 44318_2024_51_MOESM6_ESM.zip › SD Fig 5/Fig 5E/shVHL+Beclin1 WT p62.tiff]

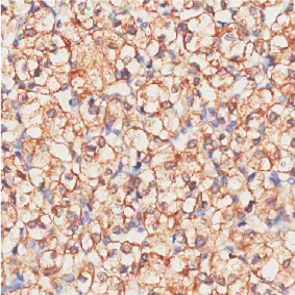

Supplement: Supplementary file 6 — Source Data Fig. 5 [file 44318_2024_51_MOESM6_ESM.zip › SD Fig 5/Fig 5E/Beclin1 WT P54-OH.tiff]

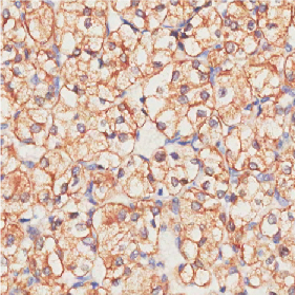

Supplement: Supplementary file 6 — Source Data Fig. 5 [file 44318_2024_51_MOESM6_ESM.zip › SD Fig 5/Fig 5E/shVHL+Beclin1 WT P54-OH.tiff]

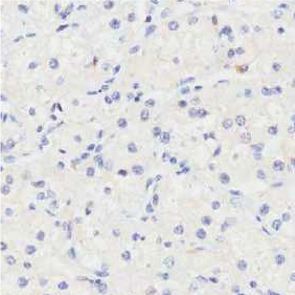

Supplement: Supplementary file 6 — Source Data Fig. 5 [file 44318_2024_51_MOESM6_ESM.zip › SD Fig 5/Fig 5E/shVHL+Beclin1 P54A P54-OH.tiff]

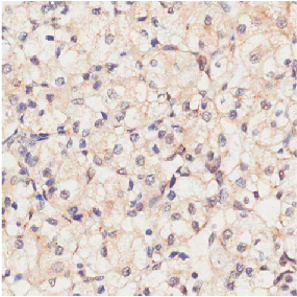

Supplement: Supplementary file 6 — Source Data Fig. 5 [file 44318_2024_51_MOESM6_ESM.zip › SD Fig 5/Fig 5E/Beclin1 P54A p62.tiff]

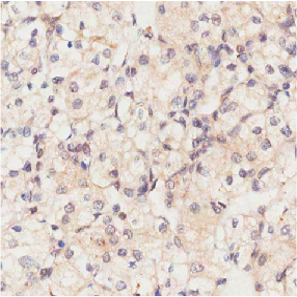

Supplement: Supplementary file 6 — Source Data Fig. 5 [file 44318_2024_51_MOESM6_ESM.zip › SD Fig 5/Fig 5E/shVHL+Beclin1 P54A p62.tiff]

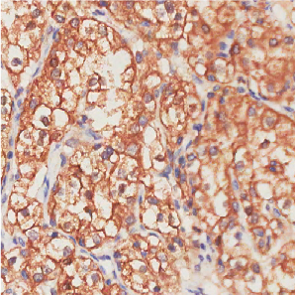

Supplement: Supplementary file 6 — Source Data Fig. 5 [file 44318_2024_51_MOESM6_ESM.zip › SD Fig 5/Fig 5E/Beclin1 Wt VHL.tiff]

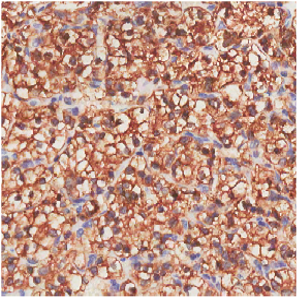

Supplement: Supplementary file 7 — Source Data Fig. 6 [file 44318_2024_51_MOESM7_ESM.zip › SD Fig 6/Fig 6A/Tumor1 LC3B.tiff]

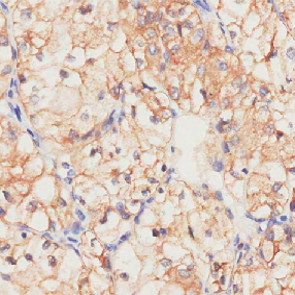

Supplement: Supplementary file 7 — Source Data Fig. 6 [file 44318_2024_51_MOESM7_ESM.zip › SD Fig 6/Fig 6A/Tumor2 VHL.tiff]

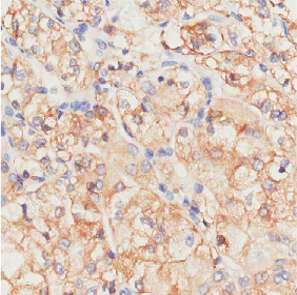

Supplement: Supplementary file 7 — Source Data Fig. 6 [file 44318_2024_51_MOESM7_ESM.zip › SD Fig 6/Fig 6A/Tumor3 VHL.tiff]

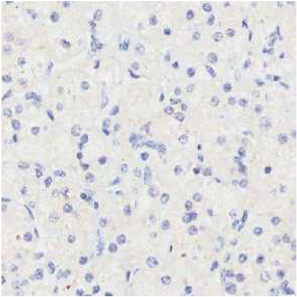

Supplement: Supplementary file 7 — Source Data Fig. 6 [file 44318_2024_51_MOESM7_ESM.zip › SD Fig 6/Fig 6A/Tumor1 P62.tiff]

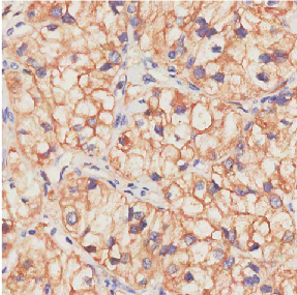

Supplement: Supplementary file 7 — Source Data Fig. 6 [file 44318_2024_51_MOESM7_ESM.zip › SD Fig 6/Fig 6A/Tumor3 P54-OH.tiff]

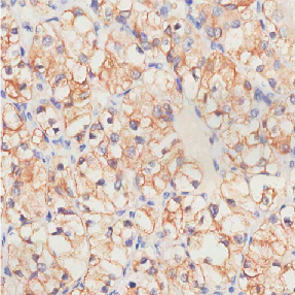

Supplement: Supplementary file 7 — Source Data Fig. 6 [file 44318_2024_51_MOESM7_ESM.zip › SD Fig 6/Fig 6A/Tumor4 VHL.tiff]

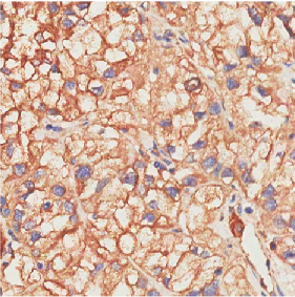

Supplement: Supplementary file 7 — Source Data Fig. 6 [file 44318_2024_51_MOESM7_ESM.zip › SD Fig 6/Fig 6A/Tumor1 Beclin1.tif]
