# Supplementary figures and images for: VHL suppresses autophagy and tumor growth through PHD1-dependent Beclin1 hydroxylation (part 2 of 2)
Source: EMBO J. 2024 Feb 15;43(6):3. doi: 10.1038/s44318-024-00051-2 (PMC10943020; doi:10.1038/s44318-024-00051-2)

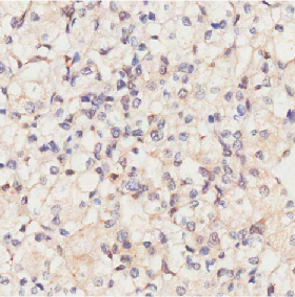

Supplement: Supplementary file 7 — Source Data Fig. 6 [file 44318_2024_51_MOESM7_ESM.zip › SD Fig 6/Fig 6A/Tumor2 P54-OH.tiff]

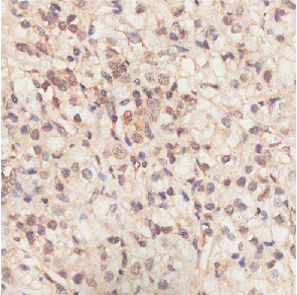

Supplement: Supplementary file 7 — Source Data Fig. 6 [file 44318_2024_51_MOESM7_ESM.zip › SD Fig 6/Fig 6A/Tumor2 P62.tiff]

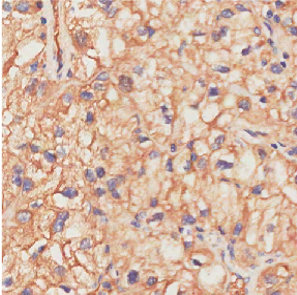

Supplement: Supplementary file 7 — Source Data Fig. 6 [file 44318_2024_51_MOESM7_ESM.zip › SD Fig 6/Fig 6A/Tumor3 P62.tiff]

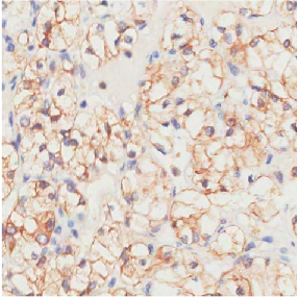

Supplement: Supplementary file 7 — Source Data Fig. 6 [file 44318_2024_51_MOESM7_ESM.zip › SD Fig 6/Fig 6A/Tumor3 LC3B.tiff]

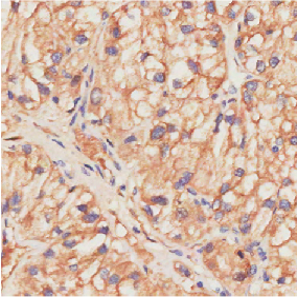

Supplement: Supplementary file 7 — Source Data Fig. 6 [file 44318_2024_51_MOESM7_ESM.zip › SD Fig 6/Fig 6A/Tumor2 Beclin1.tiff]

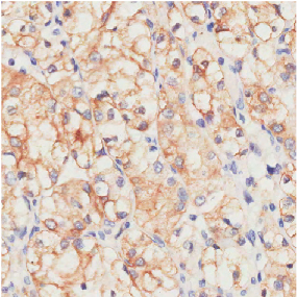

Supplement: Supplementary file 7 — Source Data Fig. 6 [file 44318_2024_51_MOESM7_ESM.zip › SD Fig 6/Fig 6A/Tumor1 VHL.tiff]

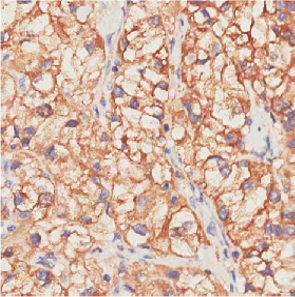

Supplement: Supplementary file 7 — Source Data Fig. 6 [file 44318_2024_51_MOESM7_ESM.zip › SD Fig 6/Fig 6A/Tumor4 Beclin1.tiff]

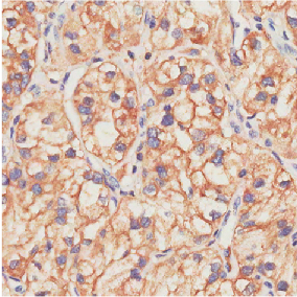

Supplement: Supplementary file 7 — Source Data Fig. 6 [file 44318_2024_51_MOESM7_ESM.zip › SD Fig 6/Fig 6A/Tumor3 Beclin1.tiff]

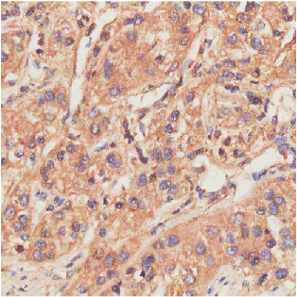

Supplement: Supplementary file 7 — Source Data Fig. 6 [file 44318_2024_51_MOESM7_ESM.zip › SD Fig 6/Fig 6A/Tumor2 LC3B.tiff]

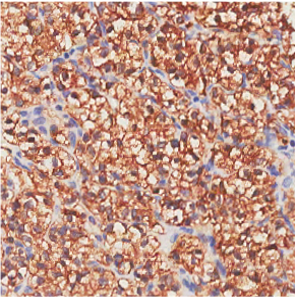

Supplement: Supplementary file 7 — Source Data Fig. 6 [file 44318_2024_51_MOESM7_ESM.zip › SD Fig 6/Fig 6A/Tumor4 P54-OH.tiff]

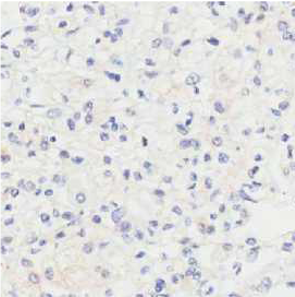

Supplement: Supplementary file 7 — Source Data Fig. 6 [file 44318_2024_51_MOESM7_ESM.zip › SD Fig 6/Fig 6A/Tumor4 LC3B.tiff]

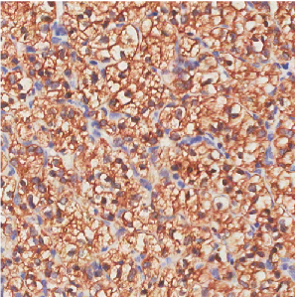

Supplement: Supplementary file 7 — Source Data Fig. 6 [file 44318_2024_51_MOESM7_ESM.zip › SD Fig 6/Fig 6A/Tumor4 P62.tiff]

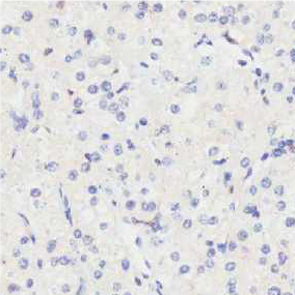

Supplement: Supplementary file 7 — Source Data Fig. 6 [file 44318_2024_51_MOESM7_ESM.zip › SD Fig 6/Fig 6A/Tumor1 P54-OH.tiff]
