# Supplementary material for: VHL suppresses autophagy and tumor growth through PHD1-dependent Beclin1 hydroxylation
Source: EMBO J. 2024 Feb 15;43(6):3. doi: 10.1038/s44318-024-00051-2 (PMC10943020; doi:10.1038/s44318-024-00051-2)
Supplement: Supplementary file 9 — Expanded View Figures [file 44318_2024_51_MOESM9_ESM.pdf]

Expanded View Figures

Figure EV1. VHL inhibits nutrient deficiency-induced autophagy in ccRCC cells in its E3 ligase activity- and HIF2α/HIF1β-independent manner.

(**A**) Flag-VHL transfected 786-O cells (left) or VHL-depleted SN12C cells (right) were treated with glucose deprivation for the indicated time. (**B**) 786-O cells stably transfected with HIF2α (left) or HIF1β (right) shRNA were treated with or without glucose deprivation for 2 h. (**C**) SN12C and TK-10 cells stably transfected with Cul2 shRNA were treated with or without glucose deprivation for 2 h. (**D**) SN12C and TK-10 cells stably transfected with VHL shRNA were harvested for immunoblotting analyses as indicated. (**E**) SN12C and TK-10 cells stably transfected with VHL shRNA were treated with CHX (100 μg/ml) for the indicated periods of time. The quantification of Beclin1 protein levels relative to tubulin levels is shown. (**F**, **G**) SN12C cells stably transfected with the indicated constructs were harvested for immunoprecipitation and immunoblotting analyses as indicated. (**H**) 786-O and RCC4 cells stably transfected with the indicated constructs were treated with or without glucose deprivation for 2 h. VPS34 complexes were immunoprecipitated by ATG14L antibody followed by PI(3)P detection by a quantitative ELISA. The PI(3)P level was normalized to the amount of ATG14L used in the assay. (**I**) 786-O cells transfected with the indicated plasmids were treated with or without glucose deprivation in the presence or absence of 20 μM chloroquine (CQ) for 2 h. (**J**) 786-O cells with or without Flag-VHL transfection were treated with amino acid (AA) deprivation for 1 h, serum deprivation for 9 h or glutamine (Gln) deprivation for 4 h, in the presence or absence of 20 μM chloroquine (CQ) for 2 h, respectively. (**K**) SN12C cells with or without VHL shRNA transfection were treated with amino acid (AA) deprivation for 1 h, serum deprivation for 9 h or glutamine (Gln) deprivation for 4 h, respectively. (**L**) SN12C and TK-10 cells were treated with or without glucose deprivation for 2 h. VPS34 complexes were immunoprecipitated by ATG14L or VHL antibodies followed by PI(3)P detection by a quantitative ELISA. The PI(3)P level was normalized to the amount of VPS34 used in the assay. Data information: Data represent the mean ± SD. The statistical significance was determined using two-tailed Student's *t* test. ***$P < 0.001$; NS no significance. (**A**–**G**, **I**–**K**) Immunoprecipitation or immunoblotting analyses were performed. (**B**, **C**, **I**, **J**) Autophagic flux were shown. All experiments were repeated three times with similar results.

© The Author(s)

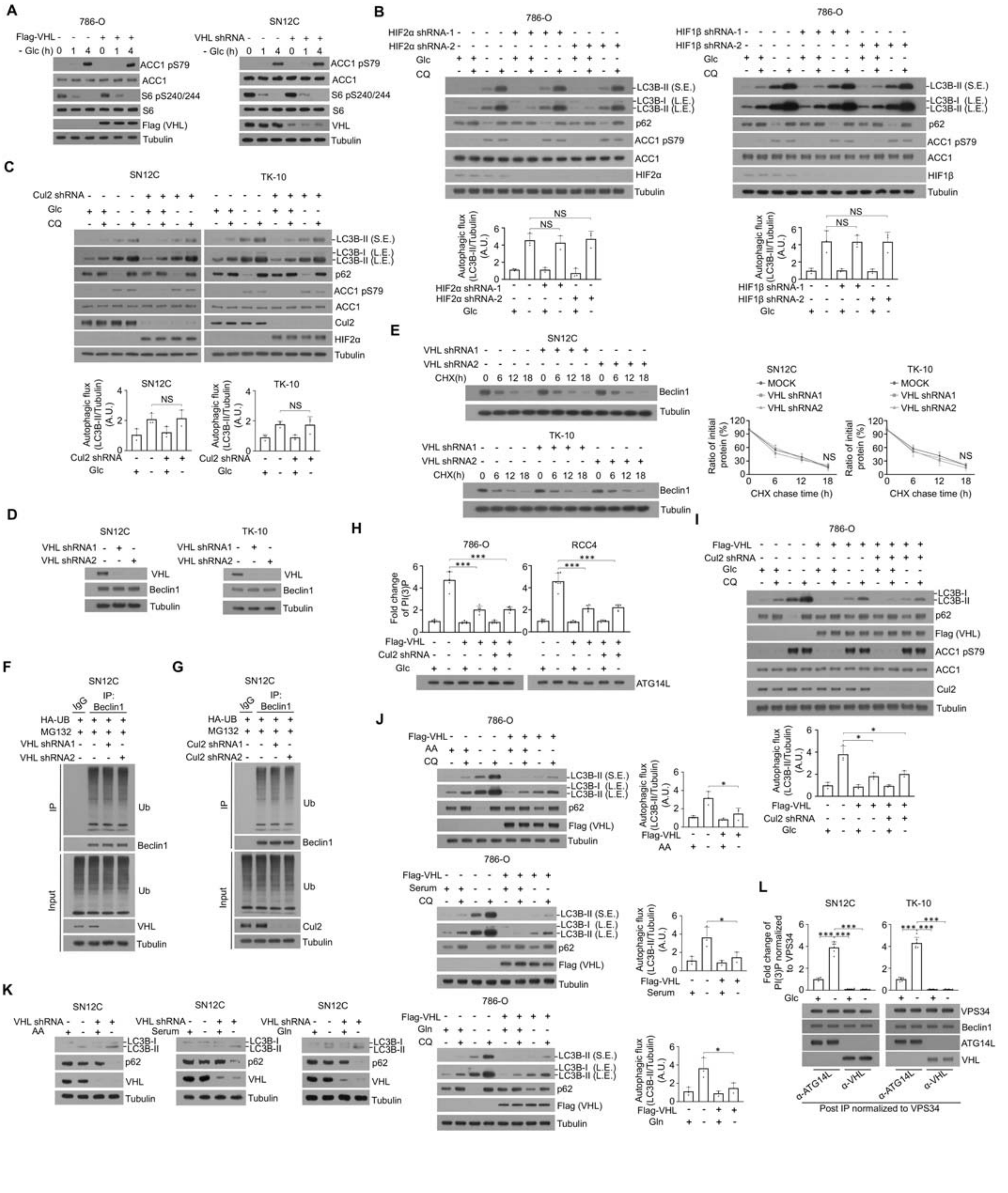

 © The Author(s)

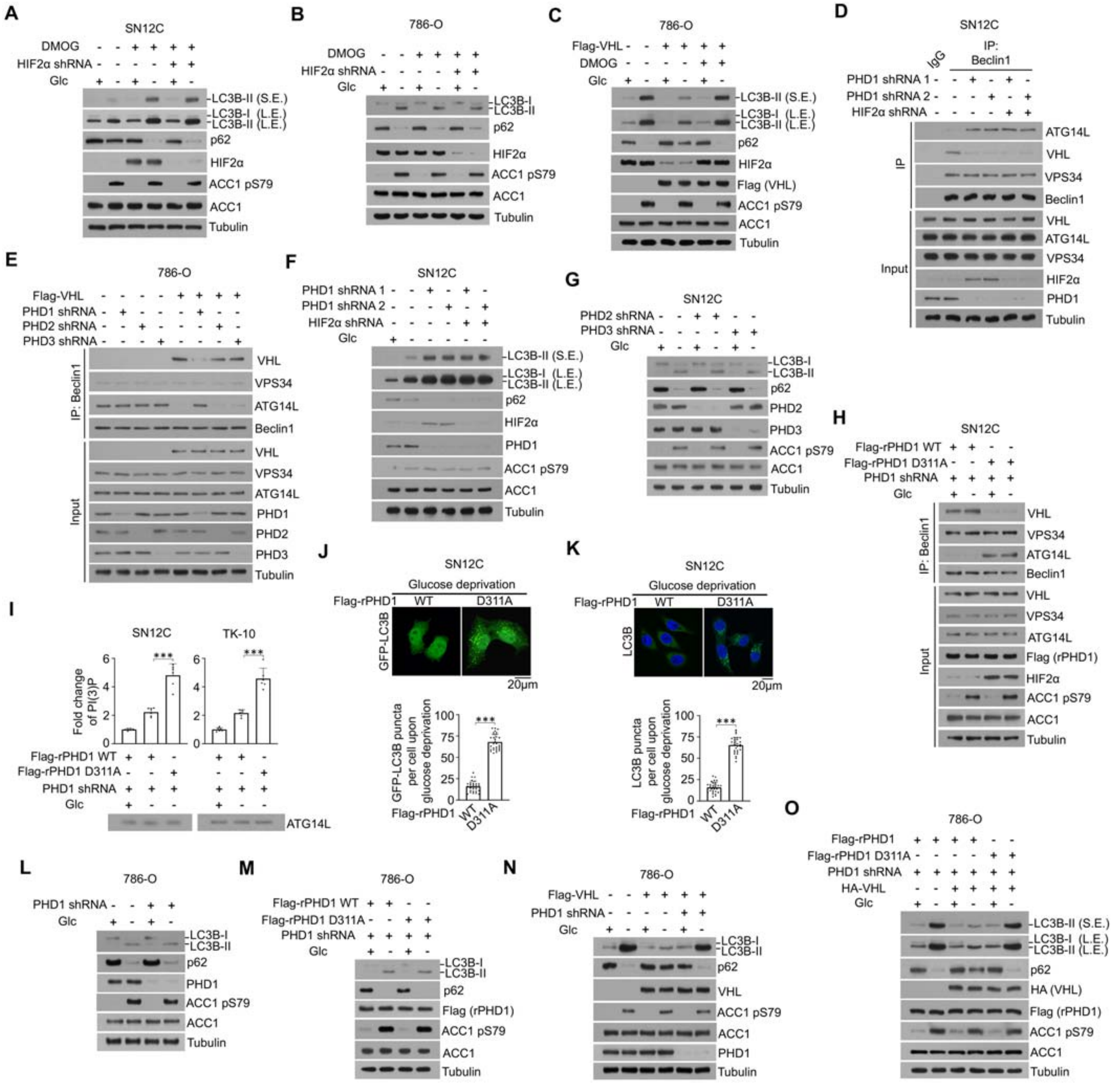
A
SN12C
DMOG
HIF2α shRNA
Glc
LC3B-II (S.E.)
LC3B-I (L.E.)
LC3B-II (L.E.)
p62
HIF2α
ACC1 pS79
ACC1
Tubulin
B
786-O
DMOG
HIF2α shRNA
Glc
LC3B-I
LC3B-II
p62
HIF2α
ACC1 pS79
ACC1
Tubulin
C
786-O
Flag-VHL
DMOG
Glc
LC3B-II (S.E.)
LC3B-I (L.E.)
LC3B-II (L.E.)
p62
HIF2α
Flag (VHL)
ACC1 pS79
ACC1
Tubulin
D
SN12C
IgG
IP: Beclin1
PHD1 shRNA 1
PHD1 shRNA 2
HIF2α shRNA
IP
ATG14L
VHL
VPS34
Beclin1
Input
VHL
ATG14L
VPS34
HIF2α
PHD1
Tubulin
E
786-O
Flag-VHL
PHD1 shRNA
PHD2 shRNA
PHD3 shRNA
IP: Beclin1
VHL
VPS34
ATG14L
Beclin1
Input
VHL
VPS34
ATG14L
PHD1
PHD2
PHD3
Tubulin
F
SN12C
PHD1 shRNA 1
PHD1 shRNA 2
HIF2α shRNA
Glc
LC3B-II (S.E.)
LC3B-I (L.E.)
LC3B-II (L.E.)
p62
HIF2α
PHD1
ACC1 pS79
ACC1
Tubulin
G
SN12C
PHD2 shRNA
PHD3 shRNA
Glc
LC3B-I
LC3B-II
p62
PHD2
PHD3
ACC1 pS79
ACC1
Tubulin
H
SN12C
Flag-rPHD1 WT
Flag-rPHD1 D311A
PHD1 shRNA
Glc
IP: Beclin1
VHL
VPS34
ATG14L
Beclin1
Input
VHL
VPS34
ATG14L
Flag (rPHD1)
HIF2α
ACC1 pS79
ACC1
Tubulin
I
SN12C
TK-10
Fold change of PI(3)P
***
Flag-rPHD1 WT
Flag-rPHD1 D311A
PHD1 shRNA
Glc
ATG14L
J
SN12C
Glucose deprivation
Flag-rPHD1 WT D311A
GFP-LC3B
20μm
GFP-LC3B puncta per cell upon glucose deprivation
***
Flag-rPHD1 WT D311A
K
SN12C
Glucose deprivation
Flag-rPHD1 WT D311A
LC3B
20μm
LC3B puncta per cell upon glucose deprivation
***
Flag-rPHD1 WT D311A
L
786-O
PHD1 shRNA
Glc
LC3B-I
LC3B-II
p62
PHD1
ACC1 pS79
ACC1
Tubulin
M
786-O
Flag-rPHD1 WT
Flag-rPHD1 D311A
PHD1 shRNA
Glc
LC3B-I
LC3B-II
p62
Flag (rPHD1)
ACC1 pS79
ACC1
Tubulin
N
786-O
Flag-VHL
PHD1 shRNA
Glc
LC3B-I
LC3B-II
p62
VHL
ACC1 pS79
ACC1
PHD1
Tubulin
O
786-O
Flag-rPHD1
Flag-rPHD1 D311A
PHD1 shRNA
HA-VHL
Glc
LC3B-II (S.E.)
LC3B-I (L.E.)
LC3B-II (L.E.)
p62
HA (VHL)
Flag (rPHD1)
ACC1 pS79
ACC1
Tubulin

Figure EV2. PHD1 is required for the binding of VHL to Beclin1 and VHL-mediated autophagy inhibition.

(**A**, **B**) SN12C (**A**) or 786-O (**B**) cells with and without HIF2α depletion were pretreated with or without DMOG (200 μM) for 1 h before glucose deprivation for 2 h. (**C**) Flag-VHL-transfected 786-O cells were pretreated with or without DMOG (200 μM) for 1 h before glucose deprivation for 2 h. (**D**, **E**) SN12C (**D**) or 786-O (**E**) cells transfected with the indicated plasmids were harvested. (**F**, **G**) SN12C cells transfected with the indicated shRNA were treated with or without glucose deprivation for 2 h. (**H**) SN12C cells with or without PHD1 depletion and reconstituted expression of the indicated shRNA-resistant Flag-PHD1 proteins were treated with or without glucose deprivation for 2 h. (**I**) PHD1 depleted SN12C or TK-10 cells with reconstituted expression of the indicated shRNA-resistant Flag-PHD1 proteins were treated with or without glucose deprivation for 2 h. VPS34 complexes were immunoprecipitated by ATG14L antibody followed by PI(3)P detection by the quantitative ELISA. The PI(3)P level was normalized to the amount of ATG14L used in the assay. (**J**, **K**) PHD1 depleted SN12C cells with reconstituted expression of the indicated shRNA-resistant Flag-rPHD1 proteins were treated with glucose deprivation for 2 h. Representative images of GFP-LC3B (**J**) or endogenous LC3B (**K**) puncta are shown (upper). The numbers of LC3B puncta from 30 cells were quantitated (lower). (**L**) 786-O cells with or without PHD1 depletion were treated with or without glucose deprivation for 2 h. (**M**) Endogenous PHD1 depleted 786-O cells with reconstituted expression of the indicated shRNA-resistant Flag-PHD1 proteins were treated with or without glucose deprivation for 2 h. (**N**) 786-O cells with or without PHD1 depletion were transfected with the indicated plasmids and treated with or without glucose deprivation for 2 h. (**O**) 786-O cells with reconstituted expression of the indicated shRNA-resistant Flag-rPHD1 proteins were transfected with the indicated plasmids and treated with or without glucose deprivation for 2 h. Data information: data represent the mean ± SD. The statistical significance was determined using two-tailed Student's *t* test. ***$P < 0.001$. (**A–H**, **L–O**) Immunoprecipitation and/or immunoblotting analyses were performed as indicated. All experiments were repeated at least twice with similar results.

 © The Author(s)

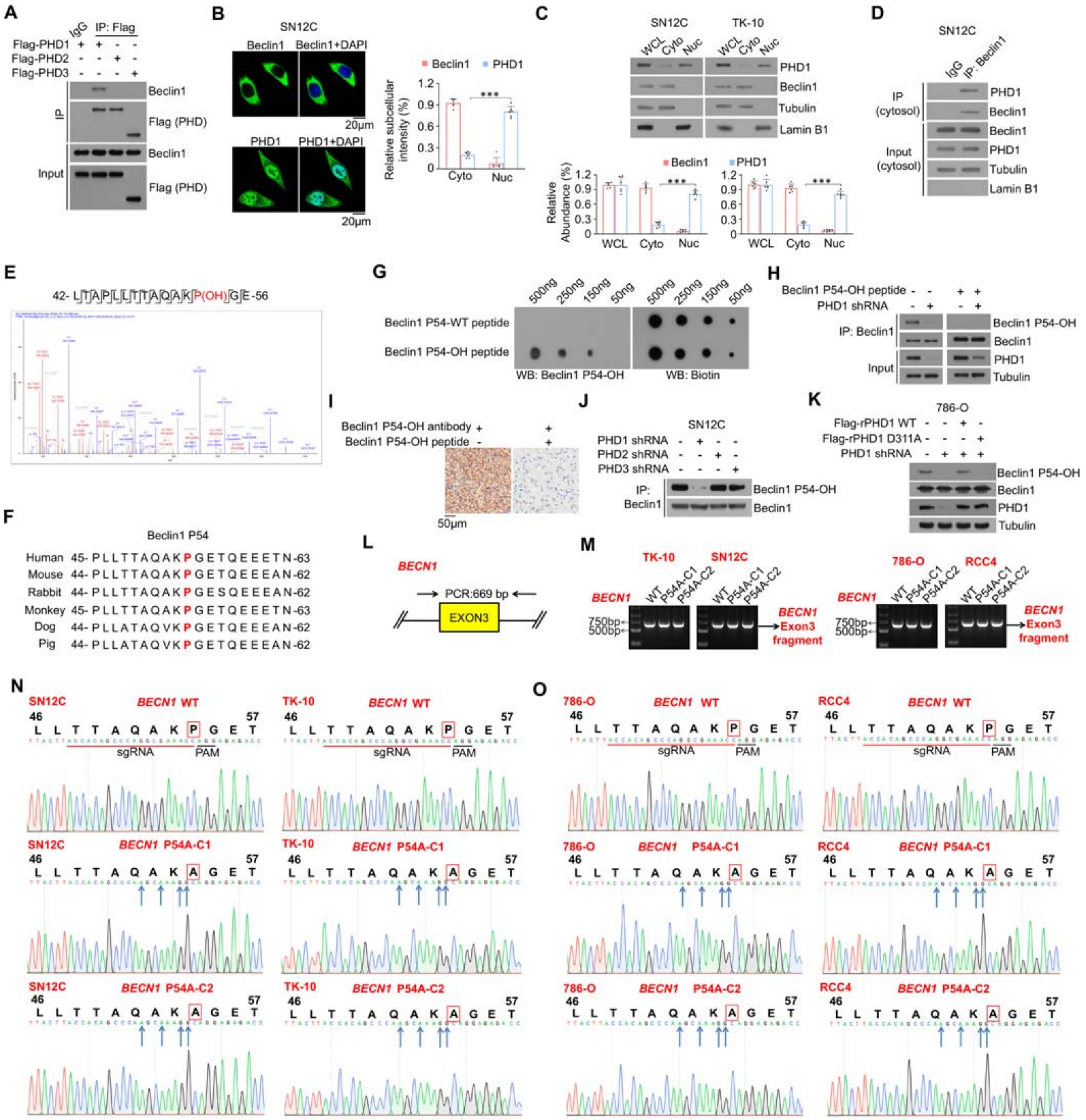
A
IgG
IP: Flag
Flag-PHD1
Flag-PHD2
Flag-PHD3
IP
Input
Beclin1
Flag (PHD)
B
SN12C
Beclin1
Beclin1+DAPI
PHD1
PHD1+DAPI
20μm
Beclin1
PHD1
Relative subcellular intensity (%)
Cyto
Nuc
C
SN12C
TK-10
WCL
Cyto
Nuc
PHD1
Beclin1
Tubulin
Lamin B1
Relative Abundance (%)
D
SN12C
IgG
IP: Beclin1
IP (cytosol)
Input (cytosol)
E
42- LTAPLLTTAQAKP(OH)GE-56
F
Beclin1 P54
Human 45- PLLTTAQAKPGETQEEETN-63
Mouse 44- PLLTTAQAKPGETQEEEAN-62
Rabbit 44- PLLTTAQAKPGESQEEEAN-62
Monkey 45- PLLTTAQAKPGETQEEETN-63
Dog 44- PLLATAQVKPGETQEEEAN-62
Pig 44- PLLATAQVKPGETQEEEAN-62
G
500ng
250ng
150ng
50ng
Beclin1 P54-WT peptide
Beclin1 P54-OH peptide
WB: Beclin1 P54-OH
WB: Biotin
H
Beclin1 P54-OH peptide
PHD1 shRNA
IP: Beclin1
Input
Beclin1 P54-OH
I
Beclin1 P54-OH antibody
Beclin1 P54-OH peptide
50μm
J
SN12C
PHD1 shRNA
PHD2 shRNA
PHD3 shRNA
IP: Beclin1
K
786-O
Flag-rPHD1 WT
Flag-rPHD1 D311A
L
BECN1
PCR:669 bp
EXON3
M
TK-10
SN12C
786-O
RCC4
WT
P54A-C1
P54A-C2
750bp
500bp
BECN1 Exon3 fragment
N
O
BECN1 WT
BECN1 P54A-C1
BECN1 P54A-C2
sgRNA
PAM
L L T T A Q A K P G E T
L L T T A Q A K A G E T

Figure EV3. PHD1 hydroxylates Beclin1 P54.

(**A**) 293T cells transfected with the indicated plasmids were harvested for immunoprecipitation and immunoblotting analyses as indicated. (**B**) Immunofluorescence analyses were performed with the indicated antibodies (left). The relative subcellular distribution intensity of Beclin1 and PHD1 is shown (right). (**C**) Cytosolic and nuclear fractions of SN12C and TK-10 cells were prepared. The relative Beclin1 and PHD1 abundance in different fractions was quantified by densitometric analysis of the blots ($n = 6$). *n* represents independent biological replicate. (**D**) The cytosolic fraction of SN12C cells were harvested for immunoprecipitation and immunoblotting analyses as indicated. (**E**) Flag/Strep Beclin1 immunoprecipitated from SN12C cells was subjected to liquid chromatography-tandem mass spectrometry/mass spectrometry (LC-MS/MS) analyses. Representative LC-MS/MS spectra showing hydroxyl-proline-containing fragments derived from Beclin1. Mass spectrometric analysis of a tryptic fragment at m/z 1391.02930 Da (+ 0.06 mmu/+0.08 ppm), which was matched with the +2 charged peptide 42-LTAPLLTTAQAKPGE-56, suggested that Beclin1 P54 was hydroxylated. The XCorr score was 4.47. (**F**) Alignment of protein sequences spanning Beclin1 Pro54 from different species. (**G**) Dot immunoblotting analyses were performed with the indicated synthetic peptides diluted with different concentrations and detected with immunoblotting analyses as indicated. (**H**) SN12C cells transfected with the indicated plasmids were harvested for immunoprecipitation and immunoblotting analyses in the presence or absence of a blocking peptide for Beclin1 P54 hydroxylation. (**I**) IHC analyses of human ccRCC samples were performed with the indicated antibodies in the presence or absence of a blocking peptide for Beclin1 P54 hydroxylation. (**J**) SN12C cells stably transfected with the indicated shRNA were harvested for immunoprecipitation and immunoblotting analyses as indicated. (**K**) 786-O cells reconstituted with the PHD1 WT or D311A mutant were harvested for immunoprecipitation and immunoblotting analyses as indicated. (**L–O**) Genomic DNA was extracted from two individual clones of different parental ccRCC cells with knock-in expression of Beclin1 P54A. PCR products were amplified from the indicated DNA fragment (**L**) and separated on an agarose gel (**M**). Sequencing of different parental ccRCC cells and two individual clones with knock-in expression of Beclin1 P54A (**N**, **O**). The red line indicates the sgRNA-targeting sequence. The black line indicates the protospacer adjacent motif (PAM). Blue arrows indicate mutated nucleotides. A mutated amino acid and its wild-type counterpart are indicated by the solid red box. Data information: Data represent the mean ± SD. The statistical significance was determined using two-tailed Student's *t* test. ****P* < 0.001. WCL whole-cell lysate, Cyto cytosol, Nuc nucleus. Experiments were repeated at least twice with similar results.

 © The Author(s)

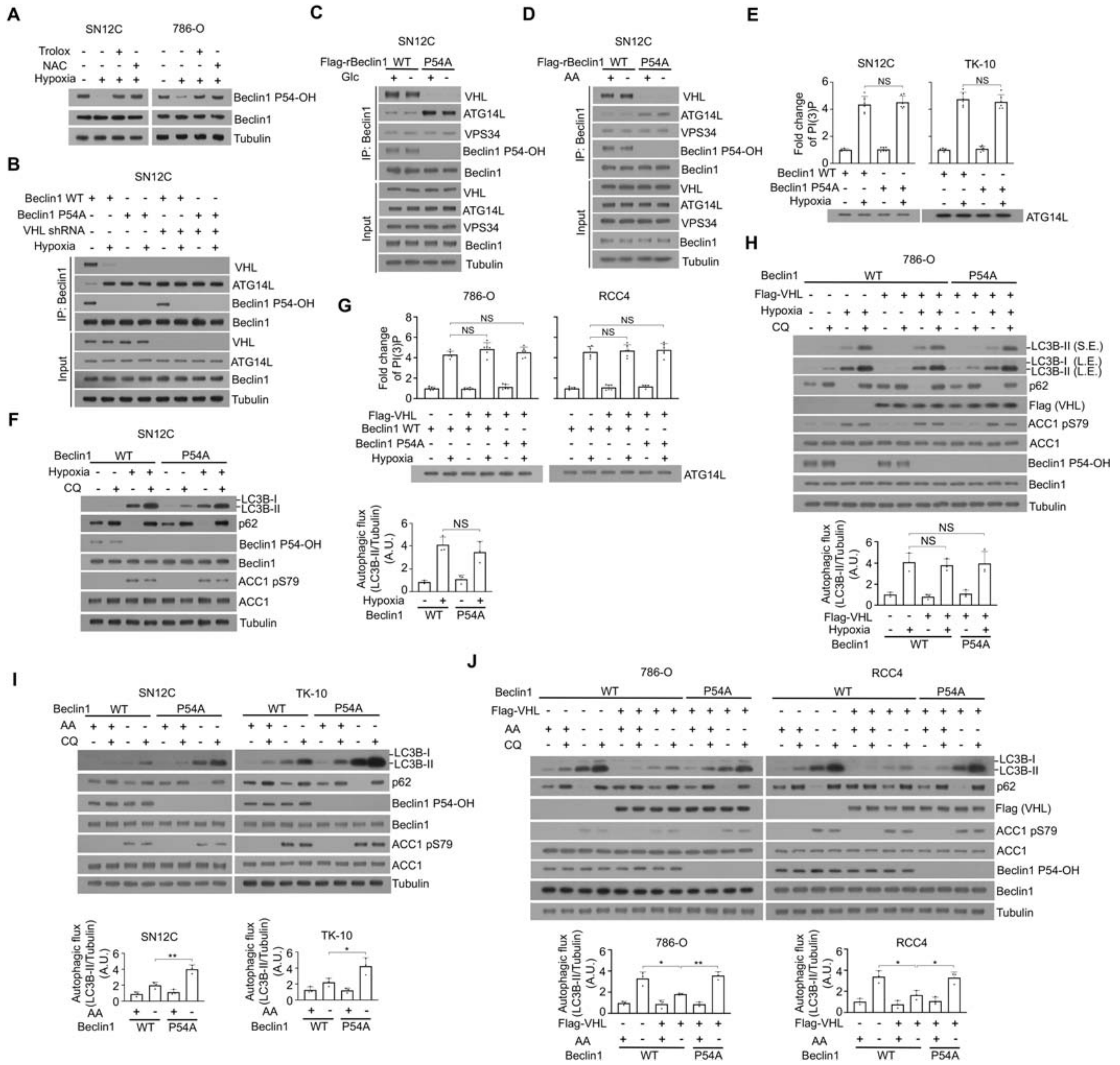
A
B
C
D
E
F
G
H
I
J
SN12C
786-O
TK-10
RCC4
Beclin1 P54-OH
Beclin1
Tubulin
VHL
ATG14L
VPS34
p62
ACC1 pS79
ACC1
Flag (VHL)
LC3B-I
LC3B-II
IP: Beclin1
Input
Fold change of PI(3)P
Autophagic flux (LC3B-II/Tubulin) (A.U.)
NS

Figure EV4. VHL suppresses autophagy in a manner depended on PHD1-mediated Beclin1 P54 hydroxylation and plays a distinct role in nutrient stress- and hypoxia-induced autophagy.

(**A**) SN12C and 786-O cells were pretreated with or without N-acetyl-L-cysteine (NAC) (5 mM) or Trolox (100 mM) for 1 h before hypoxia stimulation for 8 h. The whole-cell lysates were harvested for immunoblotting analyses as indicated. (**B**) Parental SN12C cells and the indicated clones with knock-in expression of Beclin1 P54A were stably transfected with or without VHL shRNA and treated with hypoxia for 8 h. (**C**, **D**) Endogenous Beclin1 depleted SN12C cells with reconstituted expression of the indicated shRNA-resistant Flag-Beclin1 proteins were treated with or without glucose deprivation for 2 h (**C**) or amino acid (AA) deprivation for 1 h (**D**), respectively. (**E**) Parental SN12C and TK-10 cells and the indicated clones with knock-in expression of Beclin1 P54A were treated with hypoxia for 8 h. VPS34 complexes were immunoprecipitated by ATG14L antibody followed by PI(3)P detection by quantitative ELISA. The PI(3)P level was normalized to the amount of ATG14L used in the assay. (**F**) Parental SN12C cells and the indicated clones with knock-in expression of Beclin1 P54A were treated with hypoxia in the presence or absence of 20 μM chloroquine (CQ) for 8 h. (**G**) Parental 786-O and RCC4 cells and the indicated clones with knock-in expression of Beclin1 P54A with or without Flag-VHL transfection were treated with or without hypoxia for 8 h. VPS34 complexes were immunoprecipitated by ATG14L antibody followed by PI(3)P detection by quantitative ELISA. The PI(3)P level was normalized to the amount of ATG14L used in the assay. (**H**) Parental 786-O cells and the indicated clones with knock-in expression of Beclin1 P54A were transfected with or without Flag-VHL and treated with or without hypoxia in the presence or absence of 20 μM chloroquine (CQ) for 8 h. (**I**) Parental SN12C and TK-10 cells and the indicated clone with knock-in expression of Beclin1 P54A were treated with or without amino acid (AA) deprivation in the presence or absence of 20 μM chloroquine (CQ) for 1 h. (**J**) Parental 786-O and RCC4 cells and the indicated clones with knock-in expression of Beclin1 P54A transfected with or without Flag-VHL were treated with or without amino acid (AA) deprivation in the presence or absence of 20 μM chloroquine (CQ) for 1 h. Data information: data represent the mean ± SD. The statistical significance was determined using two-tailed Student's *t* test. NS no significance. (**A–D**, **F**, **H–J**) Immunoprecipitation and/or immunoblotting analyses were performed as indicated. (**F**, **H–J**) Autophagic flux were shown. All experiments were repeated at least twice with similar results.

 © The Author(s)

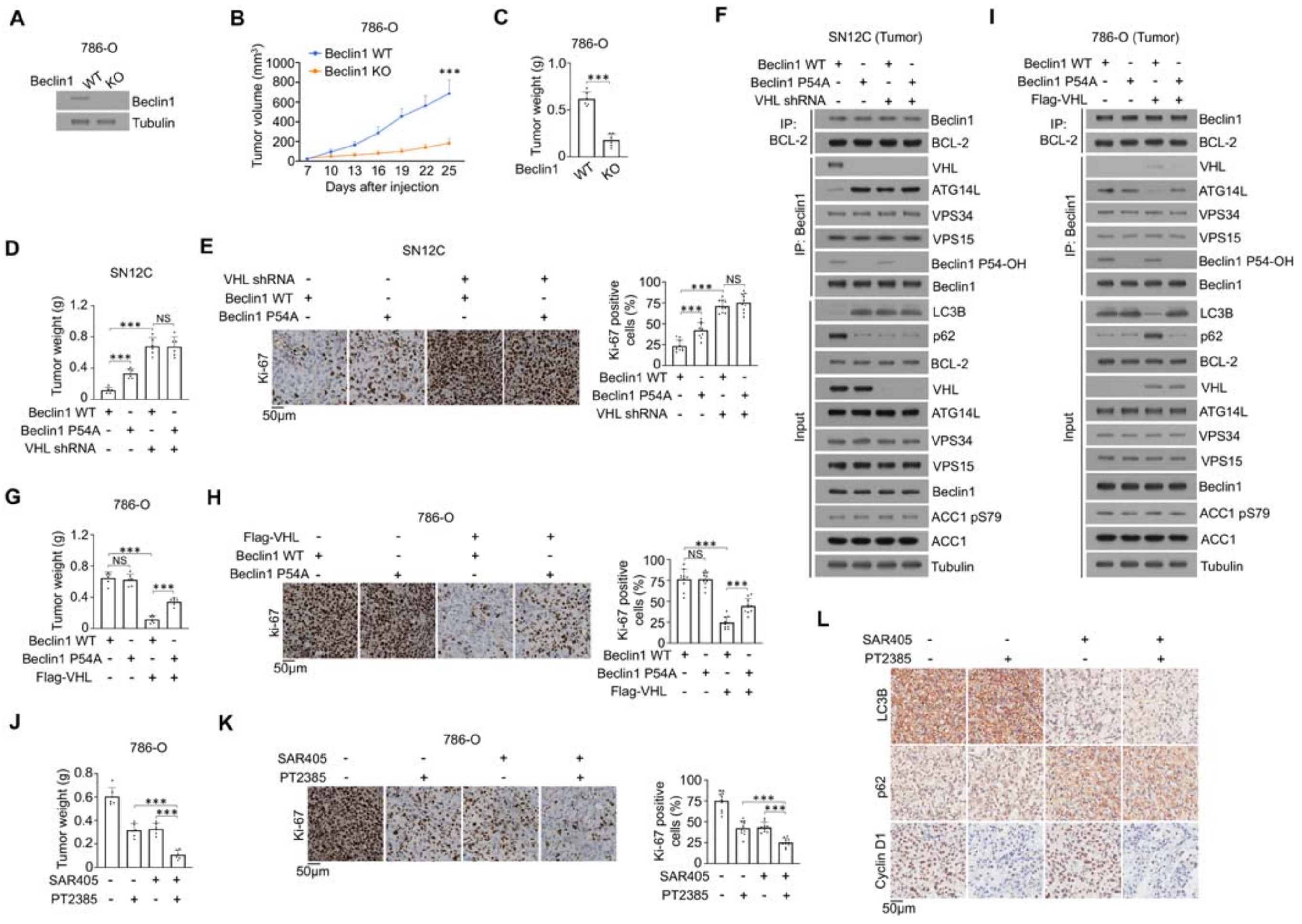

Figure EV5. PHD1-mediated Beclin1 P54 hydroxylation is required for VHL-inhibited tumor growth.

(**A–C**) The whole-cell lysates of WT and Beclin1 knockout 786-O cells were harvested for immunoblotting analyses as indicated (**A**). WT and Beclin1 knockout 786-O cells (1×10^6) were subcutaneously injected into the left or right flanks of 6-week-old male athymic nude mice ($n = 6$). n represents the number of independent animals in each group. The resulting tumors were resected 25 days after injection. Tumor volume and weight were analyzed. ***$P < 0.001$ by Student's two-tailed *t* test (**B**, **C**). (**D–F**) Parental SN12C cells (1×10^6) or the clones with Beclin1 P54A knock-in expression stably transfected with or without VHL shRNA were subcutaneously injected into the left or right flanks of athymic nude mice, respectively. The weight of xenografted tumors in the mice was measured (**D**). IHC analyses of tumor samples were performed as indicated (**E**, left). Ki67-positive cells were quantified in 10 microscopic fields (**E**, right). Immunoprecipitation and immunoblotting analyses of the indicated tumors were performed with the indicated antibodies (**F**). Data represent the mean ± SD. ***$P < 0.001$; NS no significance by Student's two-tailed *t* test. (**G–I**) Parental 786-O cells (1×10^6) and the indicated clones with Beclin1 P54A knock-in expression stably transfected with or without Flag- VHL were subcutaneously injected into the flanks of athymic nude mice, respectively. The weight of xenografted tumors in the mice was measured (**G**). IHC analyses of tumor samples were performed as indicated (**H**, left). Ki67-positive cells were quantified in 10 microscopic fields (**H**, right). Immunoprecipitation and immunoblotting analyses of the indicated tumors were performed with the indicated antibodies (**I**). Data represent the mean ± SD. ***$P < 0.001$; NS no significance by Student's two-tailed *t* test. (**J–L**) 786-O cells were subcutaneously injected into athymic nude mice. When the tumor reached 50 mm^3, the mice were assigned randomly into different treatment groups. PT2385 or SAR405 was intraperitoneally injected daily at a dose of 100 mg/kg until the endpoint at day 28. The weight of xenografted tumors in the mice was measured (**J**). IHC analyses of tumor samples were performed as indicated (**K**, left and **L**). Ki67-positive cells were quantified in 10 microscopic fields (**K**, right). Data represent the mean ± SD. ***$P < 0.001$ by Student's two-tailed *t* test.
